# Microglial Lcn2 knockout enhances chronic intracerebral hemorrhage recovery by restoring myelin and reducing inflammation

**DOI:** 10.7150/thno.109440

**Published:** 2025-03-29

**Authors:** Li Wang, Lei Zhang, Kai Wang, Jun He, Liang Yuan, Yangang Wang, Weihao Lv, Zehan Zhang, Yuan Feng, Hongchen Zhang, Min Zhang, Rui Lv, Ya-nan Dou, Xiaowei Fei, Jialiang Wei

**Affiliations:** 1 Department of Neurosurgery, Xijing Hospital, the Fourth Military Medical University, Xi'an, Shaanxi, 710032, China.; 2 ZHANG Xi Provincial Famous Traditional Chinese Medicine Studio, Affiliated Hospital of Nanjing University of Chinese Medicine, Changzhou Hospital of Chinese Medicine, Changzhou, Jiangsu, 213003, China.

**Keywords:** Lcn2, microglia, OPCs, myelin, ICH

## Abstract

**Rationale:** Damage to white matter and myelin poses a significant challenge to neurological recovery in the chronic phase of intracerebral hemorrhage (ICH). The repair of myelin damage post-ICH largely depends on the activation and differentiation of oligodendrocyte precursor cells (OPCs) into oligodendrocytes, a process that is significantly influenced by the inflammatory microenvironment. Lipocalin-2 (Lcn2) regulate phenotypic transformation of microglia and thus modulates inflammation. However, the exact role of Lcn2 in facilitating myelin recovery during the chronic phase of ICH remains to be fully understood.

**Methods:** To create the ICH model, autologous blood from male C57BL/6 and Lcn2^fl/fl^Cx3cr1^Cre^ mice was utilized. Behavioral tests were conducted to evaluate neurological recovery. The differentiation of OPCs and extent of myelin recovery were assessed using OPC and myelin markers. A multi-factor inflammatory chip was employed to investigate potential molecular regulatory mechanisms. Additionally, the Lcn2 inhibitor ZINC-94/89 was administered to explore its potential in targeting Lcn2 for enhancing myelin recovery during the chronic phase of ICH.

**Results:** Knocking out Lcn2 in microglia significantly improved behavioral performance in chronic ICH mice, reduced inflammatory response, and enhanced myelin recovery. Both *in vivo* and *in vitro* experiments confirmed that Lcn2 knockout promoted microglia transformation to the M2 phenotype and enhanced OPCs differentiation. Mechanistically, Lcn2 knockout might affect Gdf-1 secretion in BV2 cells by modulating the JAK/STAT signaling pathway. Treatment with JAK inhibitors decreased Gdf-1 expression in BV2 cells, inhibiting OPCs migration and differentiation. Additionally, phosphorylation of Stat3 at Thr705 plays a critical role in enhancing Gdf-1 transcription and translation. Administration of the Lcn2 inhibitor ZINC-94/89 significantly improved behavioral performance, reduced inflammatory response, and promoted myelin recovery in chronic ICH mice.

**Conclusions:** Lcn2 is crucial for myelin recovery in the chronic phase of ICH by modulating microglial phenotypes, thereby enhancing the migration and differentiation of OPCs. Administering an Lcn2 inhibitor early on could serve as a novel and effective strategy to boost recovery during this phase.

## Introduction

Intracerebral hemorrhage (ICH) is a major cause of disability and death in stroke patients, placing a heavy economic and emotional burden on families and society 1. Although ICH typically affects the white matter, the survival of white matter fibers after such events has not been extensively studied. Recent research highlights that damage to myelin and white matter following ICH is a crucial mechanism behind secondary brain injury 2, 3. In chronic mouse models of ICH, more than half of the subjects showed significant white matter damage in the affected brain areas. Gaining a deeper understanding of myelin regeneration and white matter injury (WMI) could provide new avenues for ICH treatment 2, 3. Myelin in the brain is primarily composed of oligodendrocytes, which originate from oligodendrocyte precursor cells (OPCs). These cells exhibit remarkable plasticity under various pathophysiological conditions, differentiating into diverse functional subgroups, migrating, and undergoing morphological and molecular changes as they mature into oligodendrocytes 4, 5. Oligodendrocytes are particularly sensitive to changes such as iron overload in the brain's microenvironment post-ICH, leading to severe damage 6. OPCs near hemorrhagic brain tissue migrate and differentiate independently of the oligodendrocyte lineage cells in the subventricular zone, suggesting that OPC activation is a key pathway for repairing oligodendrocyte damage after ICH 7. Studies have shown that OPC transplantation after spinal cord injury significantly increases the percentage of myelinated axons and stimulates functional recovery 8, 9. In a rat model of brain hypoxia, OPC transplantation has been demonstrated to induce myelination, stimulate neural stem cell proliferation, enhance spatial learning and memory recovery, and inhibit neuronal apoptosis. These findings suggest that OPC activation and differentiation may be crucial for promoting myelin regeneration and repairing damage after ICH 10, 11. Various molecules, such as platelet-derived growth factor (PDGF), vascular endothelial growth factor (VEGF), and fibroblast growth factor (FGF), have been identified as regulators of OPC migration 12, 13. However, the mechanisms governing OPC differentiation into oligodendrocytes post-ICH remain unclear.

Research indicates that OPCs function undergo significant changes in various central nervous system (CNS) diseases. Neuroinflammation activation significantly hampers the myelin repair function of OPCs; thus, targeting neuroinflammation can effectively regulate OPCs differentiation and myelin repair 14-16. However, neuroinflammatory regulation involves diverse cells and complex mechanisms, making it crucial to identify key cells involved in neuroinflammation to regulate OPC differentiation after ICH. Microglia, the primary resident immune cells in the CNS, play a key role in the central immune system 17. In the chronic phase of ICH, continuous stimulation from the hematoma and its contents leads to persistent overactivation of microglia, a key factor causing secondary damage to hemorrhagic brain tissue 18. Strategies targeting microglia regulation can effectively alleviate brain damage after ICH and improve neural function recovery. Microglia can differentiate into multiple subgroups or exhibit diverse genetic phenotypes to perform various functions under different pathophysiological conditions 19. Recent studies have found that peripheral macrophages gradually shift from a pro-inflammatory phenotype to an anti-inflammatory repair phenotype in the later stages of disease. Additionally, post-stroke brain microglia exhibit different transcriptomic expression states at various disease stages, with ICH recovery phase microglia gradually showing a pro-recovery functional phenotype 17., indicating the functional plasticity of microglia under different CNS pathological conditions. Effectively regulating the functional transformation of microglia may be key to OPCs function regulation following ICH. However, the regulatory role of such mechanisms and key molecular pathways is yet to be clarified.

Lipocalin-2 (Lcn2) is a 25 kDa member of the lipocalin family, characterized by an 8-barrel protein fold, capable of binding small hydrophobic molecules and certain larger soluble macromolecules 20. Studies show that Lcn2 is upregulated in various pathological microenvironments and involved in the pathogenesis and progression of numerous diseases, including myocardial injury post-infarction 21, non-alcoholic steatohepatitis 22, and atherosclerotic plaque formation 23. Moreover, Lcn2 is closely associated with CNS diseases like cognitive impairments and dementia 24. Research indicates that Lcn2 is a key inflammatory regulatory molecule for neuronal injury and blood-brain barrier disruption after ICH 25. Furthermore, global brain Lcn2 knockout effectively alleviates pathological changes in the brain white matter of subarachnoid hemorrhage mice, mitigates microglial dysfunction, and improves neurological outcomes, suggesting that Lcn2 might be an important regulatory molecule in myelin repair obstruction mediated by neuroinflammation post-ICH 26. However, the role and molecular mechanisms of Lcn2 in regulating microglia after ICH, and its contribution to myelin repair around hemorrhagic brain tissue, remain unclear.

In this study, we utilized transgenic mice, inflammation chip sequencing, and co-culture systems to demonstrate that knocking out Lcn2 in microglia significantly improved behavioral performance in chronic ICH mice, reduced inflammatory response, and enhanced myelin recovery. Both *in vivo* and *in vitro* experiments confirmed that Lcn2 knockout promoted microglia transformation to the M2 phenotype and enhanced OPCs differentiation. Mechanistically, Lcn2 knockout might affect Gdf-1 secretion in BV2 cells by modulating the JAK/STAT signaling pathway. Treatment with JAK inhibitors increased Gdf-1 expression in BV2 cells, promoting OPCs migration and differentiation. Additionally, phosphorylation of Stat3 at Thr705 plays a critical role in enhancing growth differentiation factor 1 (Gdf-1) transcription and translation. Administration of the Lcn2 inhibitor ZINC-94/89 significantly improved behavioral performance, reduced inflammatory response, and promoted myelin recovery in chronic ICH mice. Our findings strongly support targeting microglial Lcn2 expression as a means to improve myelin recovery, offering new hope for the treatment of chronic ICH patients.

## Materials and methods

### Animals

All animal experiments were performed in accordance with protocols approved by the Institutional Ethics Committee of Xijing Hospital. All experimental procedures were approved by the Institutional Animal Care and Use Committee of Air Force Military Medical University (IACUC: 20210424). The ARRIVE 2.0 guidelines were followed for animal data report. Male C57BL/6J, Cx3cr1^Cre^Lcn2^fl/fl^, GFAP^Cre^Lcn2^fl/fl^ and Nestin^Cre^Lcn2^fl/fl^ mice were purchased from Cyagen Biotechnology Co., Ltd (Jiangsu, China). All mice were maintained in the same environment.

### ICH model

The ICH model was established as previously described with minor modifications 27. The mice were anesthetized with xylazine (5 mg/kg) and ketamine (90 mg/kg) injected intraperitoneally. The rectal temperature was maintained at 37.5 °C. A stereotactic technique was used to make a scalp incision along the midline and a burr hole was drilled on the left side of the skull (0.2 mm anterior and 2.5 mm lateral to the bregma). Thirty microliters of autologous blood obtained from the femoral artery were transferred into a 50 μL Hamilton syringe. The syringe was connected to a microinjection pump and the needle was inserted into the brain through the burr hole (depth, 3.5 mm from the bone surface). Thirty microliters of autologous blood were injected within 10 min. The syringe was withdrawn after 10 min. After surgery, the skull hole was sealed with bone wax and the incision was closed with sutures. To avoid postsurgical dehydration, normal saline (0.5 mL) was subcutaneously injected into each mouse immediately after the surgery.

One week after ICH in mice, the disease enters a chronic phase. However, studies indicate^28^, ^29^ that the recovery and regeneration of myelin are most active during the fourth week post-injury. The 30-day time point is generally considered a critical period for observing myelin recovery, as the processes of neuronal and myelin regeneration may have begun by this time but are not yet fully completed. Therefore, we chose day 30 post-ICH as the time point for pathological and molecular analyses. In addition, because the water maze test requires seven consecutive days and to avoid the behavioral test procedures affecting the mice, we housed them for an additional three days after the behavioral tests to ensure the subsequent examinations were reliable and valid. Thus, we started the orientation navigation test on day 21, conducted probe test and other behavioral tests on day 28, and carried out the molecular and pathological assessments on day 30.

### Extraction and cultivation of primary neurons

Neonatal C57BL/6 J mice were used to extract primary neurons using a stereomicroscope. The culture dish was coated with 0.2 mg/mL of poly-L-lysine (Sigma-Aldrich) overnight at 37 °C, washed three times with sterile water and placed in an incubator for use. The brain tissue was minced with sterile ophthalmic scissors, digested with 0.25% trypsin for 5 min at 37 °C before the brain tissue was centrifuged at 1000 rpm for 5 min. For the extraction of primary neurons, the complete Dulbecco's modified Eagle's medium (DMEM, Gibco) was used for appropriate dilution and the cell suspension was made into a seed plate. After 4-6 h, DMEM was replaced with Neurobasal medium (Gibco) for primary neurons which containing 0.25% glutamine (Sigma- Aldrich), 1% penicillin/streptomycin (Gibco) and 2% B-27 supplement (Gibco). Neurons monolayers were obtained at the bottom of the dish after 7 days. The neurons were identified by morphological analysis and neuron specific enolase (NSE) staining.

### Extraction and cultivation of primary OPCs

Add poly-L-lysine solution (100 μg/mL) to coat the cell culture flask, ensuring it completely covers the bottom of the flask. Place it in a 37 °C incubator to coat overnight. Wash three times with sterile ddH2O and air-dry before use. Neonatal mouse brain tissue is used for primary cell extraction. Rinse the isolated tissue three times with pre-cooled HBSS and gently triturate to form a cell suspension. Then add 0.25% trypsin solution and digest in a 37 °C incubator for 10 min. Add fetal bovine serum to stop the digestion and gently pipette until a single-cell suspension is formed. Filter the cell suspension through a 70 μm cell strainer and collect the cells passing through the strainer. Centrifuge at 1000 rpm for 5 min, remove the supernatant, and resuspend in DMEM + 10% FBS culture medium. Change the medium once after 24 h and then every 3 days. Once the mixed glial cells are confluent by day 8, place the culture flask on a shaker at 220 rpm in a 37 °C incubator for 1-1.5 h. After changing the medium, continue shaking at 220 rpm overnight at 37 °C. Collect the shaken medium, centrifuge at 100 g for 10 min, and remove the supernatant to obtain cell clusters of OPCs at the bottom. After counting, these can be used for co-culture experiments or resuspended in OPC proliferation medium: Neurobasal + 2% B27 + 10 ng/mL bFGF + 10 ng/mL PDGF-AA + 2 mmol/L glutamine, seeding at a density of 1×10^4/cm². Flow cytometry and WB is used to detect OPCs-specific surface antigens (PDGFRα, NG2, A2B5, MAG, PLP and MBP) for OPCs differentiation status detection, and markers CD133 for OPCs stemness detection.

### The ICH cell model

The ICH cell model was established as previously described 30. Cells were treated with erythrocyte lysate (1 μL of red blood cell lysate per mL of medium) to create an *in vitro* ICH inflammation model. The cells were incubated for different durations and used in different experiments. Erythrocyte lysates were prepared with red blood cell lysis buffer (Solarbio, Beijing), and the experimental process was in strict accordance with the manufacturer's instructions.

### Mouse genotype detection

Mouse tails were cut, digested with proteinase K for 20 min at 55 °C, and further inactivated with proteinase K for 5 min at 100 °C. Polymerase chain reaction (PCR) was performed according to the protocol of the One Step Mouse Genotyping Kit (Vazyme, China). Lcn2 primers: F: 5'-TGA TCA TTC TGT GTC CTA GGG GAT G-3', R: 5'-TTA GCC TCT TCC AAG GCT AGA CAA-3' (Homozygotes: one band with 203 bp, Heterozygotes: two bands with 203 bp and 144 bp, Wildtype allele: one band with 144 bp). GFAP-Cre primers: F: 5'-TAG CCC ACT CCT TCA TAA AGC CCT-3', R: 5'-GCT AAG TGC CTT CTC TAC ACC-3' (Wildtype: N.A. Targeted: 700 bp). Cx3cr1-Cre primers: F1: 5'-GAC ATT TGC CTT GCT GGA C-3', R: 5'-GCA GGG AAA TCT GAT GCA AG-3' (Wildtype: N.A. Targeted: 380 bp). Nestin-Cre primers: P1: 5'-TTG CTA AAG CGC TAC ATA GGA-3', P2: 5'-GCC TTA TTG TGG AAG GAC TG-3', P3: 5'-CCT TCC TGA AGC AGT AGA GCA-3' (Mutant: 150bp, Wild type: 246bp).

### Enzyme-Linked Immunosorbent Assay (ELISA)

Cell supernatants from the different treatment groups were harvested for ELISA. Mice were anesthetized at different time points after ICH induction, and the brain tissue around the bleeding site was used for ELISA. ELISA was performed in strict accordance with the manufacturer's instructions. The following ELISA kits were used for detection: Mouse IL-1 beta ELISA Kit (Abcam, UK), Mouse TNF alpha ELISA Kit (Abcam, UK), Mouse IL-18 ELISA Kit (Abcam, UK), Mouse IL-4 ELISA Kit (Abcam, UK) and Mouse IL-10 ELISA Kit (Abcam, UK).

### Open field test

The open field test was performed in a quiet environment and each experiment was completed in the same period. The open field box of the mice was 30 cm high, with a 75 cm bottom edge length and a white bottom surface. Open field experiments were performed sequentially according to the group and number of mice. The animals were placed inside the box at the center of the bottom surface and photographed and timed simultaneously. Shooting was stopped after 5 min of observation, and the inner wall and bottom of the square box were cleaned (75% alcohol solution) to avoid information left by the last animal (such as urine and olfactory cues) from affecting the subsequent test results. OpenField 2.8.5 software (Mobiledatum Co., Ltd, Shanghai, China) was used to calculate the total movement distance and the movement distance within the central area.

### Rota rod system

The mice were trained before the rotating rod experiment. Each mouse was trained three times a day for 5 min, with an interval of 15 min. During the training, the rotating speed of the stick is 20 rpm. On the fourth day, the rotating rod experiment was carried out on each mouse. The equipment parameter (zhongshidichuang Science, Beijing; ZS-RDM) is set to accelerate the speed from 0 to 60 rpm within 5 min. The end of the experiment was regarded as the mouse falling from the rotating rod or holding the rotating rod for 3 turns, and the time from the beginning to the end was recorded.

### Morris water maze (MWM)

The pool diameter for the MWM experiment was 120 cm (zs-001, Zhongshidichuang Science and Technology Development Co., Ltd, Beijing). The platform was in the fourth quadrant (8 cm diameter). The pool was filled in advance, and the water surface was placed 1 cm above the platform. Titanium dioxide was added before training and mixed well to make the pool opaque. The water temperature was controlled at 20-22 °C. Orientation navigation tests were performed sequentially according to the group and number of mice. The latency time was recorded, and if the latency time did not reach 60 s, the operator was informed to guide the mouse onto the platform and to make it remain on the platform for 20 s so that it could become familiar with the surrounding environment before being removed; if the mouse swam to the platform within 60 s latency time and stayed on the platform for 5 s, the mouse was allowed to remain on the platform for another 15 s to become familiar with the surrounding environment before being removed. After all mice completed each round of testing, the next round was performed at intervals of more than 30 min for a total of four training sessions per day. Training was performed for six days. The probe test was started 24 h after completion of the orientation navigation test. The platform was removed from the pool after all mice completed the first round of testing, then a second test was performed at 2 h intervals. The direction of water entry was consistent between the two tests, and the method was the same as the orientation navigation test. The Labmaze V3.0 animal behavioral trajectory analysis system was used to analyze the escape latency of mice, the percentage of residence time in the platform quadrant, and the number of crossing platform positions.

### Quantitative polymerase chain reaction (qPCR)

Primary astrocytes were harvested for RNA extraction after different treatments using TRIzol reagent. Mice were anesthetized at different time points after ICH induction, and the brain tissue around the bleeding site was used for qPCR. Reverse transcription was performed according to the protocol of the HiScript II Q Select RT SuperMix for qPCR (+gDNA wiper) kit (Vazyme, China). qPCR was performed according to the protocol of the ChamQ SYBR Color qPCR Master Mix (Low ROX Premixed) kit (Vazyme, China). The primer information for mRNA can be found in the supplementary materials Table S1.

### Immunofluorescence (IF), western blot (WB), flow cytometry and transmission electron microscopy (TEM)

IF, WB, flow cytometry and TEM were performed as previously described 27, 31-33. The following antibodies were used: Anti-MBP (Abcam, ab7349), Anti-MAG (Abcam, ab277524), Anti-NF200 (Proteintech, 18934-1-AP), Anti-β-actin (Abcam, ab8226), Anti-NeuN (Abcam, ab177487), Anti-Iba-1 (Abcam, ab178846), Anti-iNOS (Abcam, ab283655), Anti-Lcn2 (Abcam, ab216462), Anti-PDGFRα (Abcam, ab203491), Anti-NG2 (Abcam, ab275024), Anti-PLP (Abcam, ab254363), Anti-A2B5 (Millipore Sigma, MAB312R), Anti-MAP2 (Abcam, ab183830), Anti-CD133 (Abcam, ab271092), Anti-CD206 (BioLegend, 141708), Anti-Gdf-1 (Biorbyt, orb522485), Anti-P-Jak3Tyr980/981 (CST, 5031), Anti-Jak3 (CST, 8827), Anti-P-Stat3 Tyr705 (CST, 9145), Anti-Stat3 (CST, 9139) and Anti-H3 (CST, 9715).

### Luxol Fast Blue (LFB) staining

Begin with formalin fixed. Cut the tissue into 5-10 µm thick sections using a microtome and mount them onto glass slides. Place the slides in an oven at 60 °C for 30 min to melt the paraffin. Then, deparaffinize the sections by immersing them in xylene for 2 x 5 min, followed by a series of graded ethanol solutions (100%, 95%, 70%) for 5 min each to rehydrate the tissue. Rinse the slides in distilled water for 5 min to remove any residual ethanol. Prepare a 0.1% Luxol Fast Blue solution in 95% ethanol. Stain the sections in this solution for 1-2 h at 60 °C. The staining time may vary depending on the tissue type and desired intensity. After staining, differentiate the sections in 0.05% lithium carbonate solution for 30 s to 1 min. Rinse the sections in distilled water and then counterstain with 0.1% cresyl violet solution for 5-10 min to visualize cell nuclei. Dehydrate the sections through a series of graded ethanol solutions (70%, 95%, 100%) for 5 min each, followed by two washes in xylene for 5 min each. Finally, mount the slides with a suitable mounting medium and cover with a coverslip. Examine the stained sections under a light microscope. Myelin will appear blue, while other cellular components will be stained according to the counterstain used.

### Lentivirus and plasmids

Cells were infected with lentivirus to stably knock-down Lcn2 (Lcn2-KD) (LV-Ubi-shRNA-Lcn2-3FLAG-SV40-EGFP-IRES-puromycin). The lentivirus was constructed with the assistance of GeneChem Co., Inc. (Shanghai, China). Lcn2 and Stat3 were knocked out using CRISPR/Cas9 technology. Cas9 and single guide RNA (sgRNA) lentiviruses were designed and constructed by GeneChem Co., Ltd. (Shanghai, China). Cell lines were screened with puromycin.

Stat3 overexpression plasmid, Stat3 mutant plasmid (Tyr705Ala), Luciferase reporter plasmid (RV-Gdf-1), pcDNA3.1 and negative control (NC, Luciferase reporter plasmid without promoter) were designed and constructed by Hanbio Co., Ltd. (Shanghai, China). Plasmid transfection was performed using jetPRIME DNA transfection reagent (PolyPlus) according to the manufacturer's instructions.

### Mouse cytokine array

The brain tissue around the bleeding site was used for the detection of inflammatory factors and cytokines. The mouse cytokine L308 array was purchased from Ray- Biotech (AAM-BLG-1-8; USA). The experimental procedures were performed in strict accordance with the manufacturer's instructions.

### Dual-luciferase reporter assay

Gdf-1 firefly luciferase and Renilla reporter plasmids and Stat3 overexpression plasmid were transfected into each modified cell line. The cells were cultured for 24h after plasmid transfection, and the fluorescence intensity of each treatment group was detected using the Dual-Luciferase Reporter Assay Kit (Promega).

### Data processing

For molecular biology experiments, three technical replicate experiments were performed for each mouse and the data were averaged. The three mean values (n = 3/group) obtained from three mice in each group were used for statistical comparisons between groups. *In vitro* experiments, assays were performed on cells in three wells for each experiment to obtain an average count, and in three independent biological replicates.

For pathological experiments, one section from each of three mice in each group was observed under immunofluorescence laser confocal microscopy, and three fields randomly selected from each section were used to quantify the detection indicators and the data were averaged. The three mean values (n = 3/group) obtained for each group were used for statistical comparisons between groups.

### Statistical analysis

Prism 8 for macOS software was used for the statistical analyses. PASS software was used to perform power analysis, ensuring an appropriate sample size. The power value greater than 0.9 was considered indicative of an adequate sample size in the experimental design. All values for each group are presented as mean ± SD. Parametric and nonparametric tests were used according to the homogeneity of variance. According to different comparison situations, statistical differences were analyzed using Student's t-test or one-way ANOVA, as appropriate, with Sidak's or Turkey's multiple comparisons test. P<0.05 indicated that the difference was statistically significant.

## Results

### Knockout Lcn2 in microglia significantly enhances behavioral performance and reduces inflammatory responses in mice during the chronic phase of ICH

**Figure 1:**
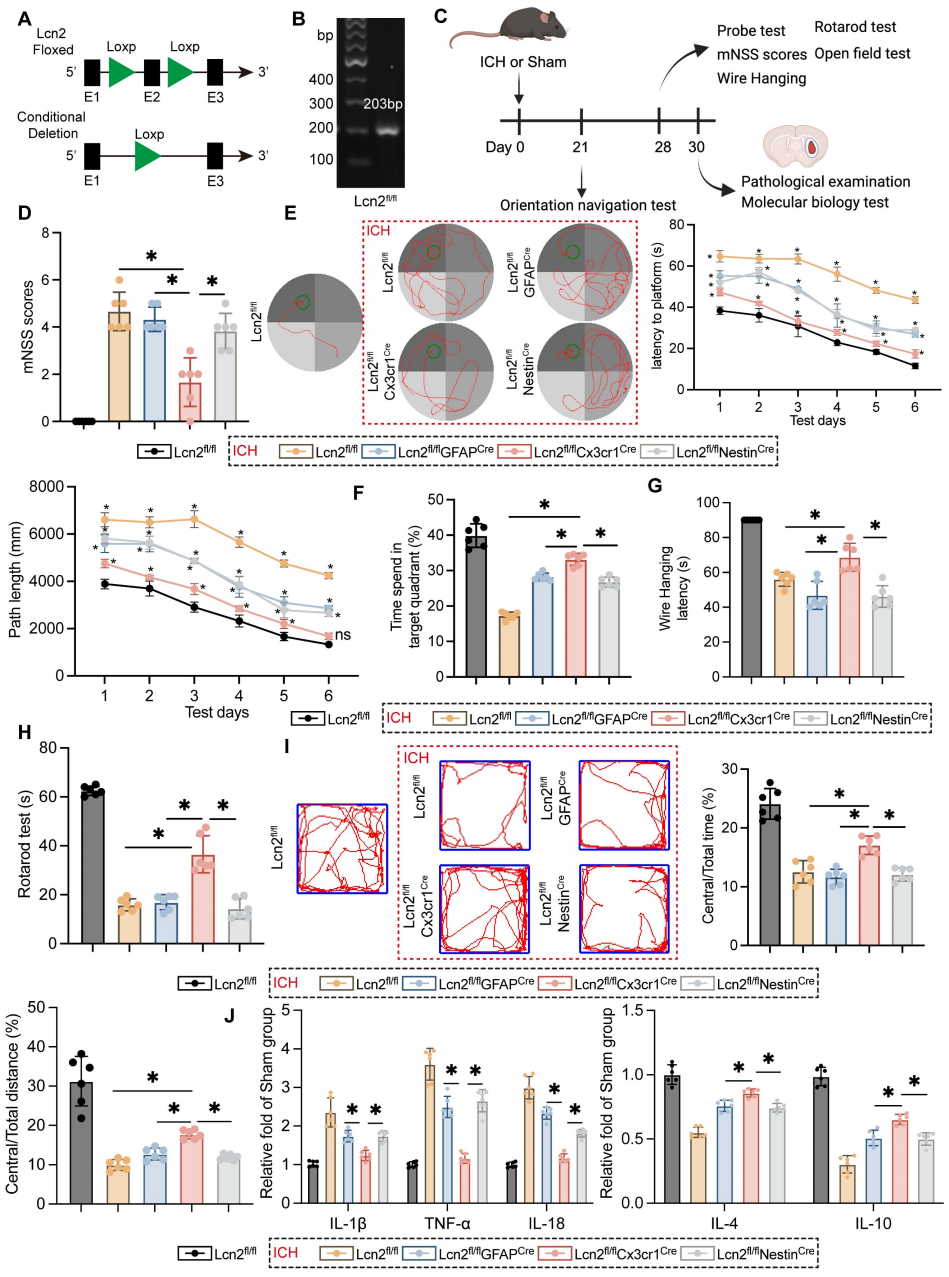
Knocking out *Lcn2* gene in microglia effectively improves the behavioral manifestations and inflammatory response of chronic ICH in mice. **A** Schematic diagram of Lcn2 fl mouse construction.** B** Identification of Mouse Tail Genes.** C** Time points for mouse modeling and various detection experiments. **D** mNSS scores F (4, 25) = 46.69, *P < 0.0001*.** E** Representative trajectory diagram of the sixth day of orientation navigation phase and quantization in the MWM. Latency: F_Interaction_ (20, 150) = 6.558, *P < 0.0001.* Path length: F_Interaction_ (20, 150) = 5.737, *P < 0.0001.* **F** Quantization of result in the probe test in MMW F (4, 25) = 116.5, *P < 0.0001*. **G** Quantify the latency of the Wire Hanging experiment F (4, 25) = 54.73 *P < 0.0001*.** H** Quantify of rotarod test F (4, 25) = 136.5, *P < 0.0001*.** I** Trajectory diagram and quantitative results of mice in open field experiment. Central/Total time: F (4, 25) = 53.03, *P < 0.0001*. Central/Total distance: F (4, 25) = 48.60, *P < 0.0001*. **J** ELISA was detected the expression levels of relevant inflammatory factors. IL-1β: F (4, 25) = 39.08, *P < 0.0001*. TNF-α: F (4, 25) = 102.2, *P < 0.0001*. IL-18: F (4, 25) = 151.9, *P < 0.0001*. IL-4: F (4, 25) = 72.62, *P < 0.0001*. IL-10: F (4, 25) = 113.0, *P < 0.0001*. The data were analyzed using one-way (**D**, **F, G**, **H**, **I** and **J**) or two-way (**E**) analysis of variance and all data are expressed as the mean ± standard deviation. *P < 0.05 represents a statistically significant difference between the two groups. ns: no statistical difference.

To explore the impact of Lcn2 during the chronic phase of ICH in mice, Loxp sites were inserted at exon 2 of Lcn2, employing the Loxp/Cre system to achieve conditional knockout in various cell types (Figure 1A). The genetically modified mice were verified as homozygous through PCR genotyping (Figure 1B). Following ICH induction, the mice were maintained on a standard diet for four weeks and underwent pathological, molecular biological, and behavioral assessments at designated time points according to the experimental protocol (Figure 1C). Compared to Lcn2 knockout in astrocytes and neurons, microglial Lcn2 knockout significantly improved the modified neurological severity score (mNSS) during the chronic phase of ICH (Figure 1D). In the MWM test, microglial Lcn2 knockout effectively reduced both the latency to reach the platform and the path length during the orientation navigation phase (Figure 1E). Additionally, in the probe test, microglial Lcn2 knockout resulted in the longest duration spent in the target quadrant compared to other cell-type knockouts (Figure 1F). Results from the Wire Hanging (Figure 1G) and Rotarod tests (Figure 1H) indicated that Lcn2^fl/fl^Cx3cr1^Cre^ mice exhibited superior grip strength, balance, and endurance during the chronic phase of ICH. The open field test further revealed that Lcn2^fl/fl^Cx3cr1^Cre^ mice had the highest proportion of distance traveled and time spent in the central area compared to other transgenic mice. Moreover, we assessed inflammatory cytokines at the hemorrhage site during the chronic phase of ICH. ELISA results showed that while Lcn2^fl/fl^GFAP^Cre^ and Lcn2^fl/fl^Nestin^Cre^ mice exhibited decreased expression of IL-1β, TNF-α and IL-18, and increased expression of the anti-inflammatory cytokine IL-4 and IL-10, the changes in inflammatory cytokines were more pronounced and significant in Lcn2^fl/fl^Cx3cr1^Cre^ mice (Figure 1J). However, knocking out Lcn2 in any type of cell does not affect the occurrence of chronic cerebral edema in mice during ICH (Figure S1A). In addition, we also examined the long-term effects of Lcn2 gene knockout on brain function. Behavioral results from various tests in wild-type (WT) mice and Lcn2 knockout mice (Lcn2^fl/fl^Cx3cr1^Cre^) of different ages showed that, regardless of whether the mice were 10 months (middle-aged) or 18 months (elderly) old, Lcn2 knockout did not have a significant impact on learning, memory, motor skills, balance, or emotional depression compared to WT mice. Most importantly, the results of the two-way ANOVA for the behavioral experiments indicated that there is no interaction between age and gene knockout as factors (Figure S1B-F). These data suggest that Lcn2 gene knockout does not have a significant effect on brain function across different age groups of mice.

OPCs, as key cells in myelination, play a crucial role in assessing the recovery levels of ICH mice one-month post-surgery when Lcn2 is knocked out in OPCs. Due to the lack of specific Cre mice for knocking out Lcn2 in OPCs, AAVs were utilized to target and intervene in the expression of Lcn2 in OPCs. The aforementioned indicators were also measured to evaluate efficacy, and the results indicated that knocking out Lcn2 in OPCs had no effect on mNSS scores (Figure S2A). The orientation navigation experiment showed significant differences between groups only on the third day of training (Figure S2B). Results from the probe test, wire hanging, and rotarod test indicated that knocking out Lcn2 in OPCs had therapeutic effects. However, the quantified values of these indicators were all lower than those in mice with Lcn2 knocked out in microglia (Figure S2C-E). Unfortunately, the open field test yielded negative results (Figure S2F). Inflammatory marker analysis revealed significant intergroup differences only for IL-1β, TNF-α, IL-4, and IL-10 (Figure S2G). The data suggest that Lcn2 may primarily function in microglia rather than in OPCs.

Altogether, although knocking out Lcn2 in neurons or astrocytes also improved ICH prognosis, the effect was not as pronounced as it was in the microglia-specific Lcn2 knockout. These findings suggest that microglial Lcn2 knockout effectively enhances behavioral performance and mitigates inflammatory responses in mice during the chronic phase of ICH.

### Microglial Lcn2 knockout enhances neuronal myelin recovery during the chronic phase of ICH

**Figure 2:**
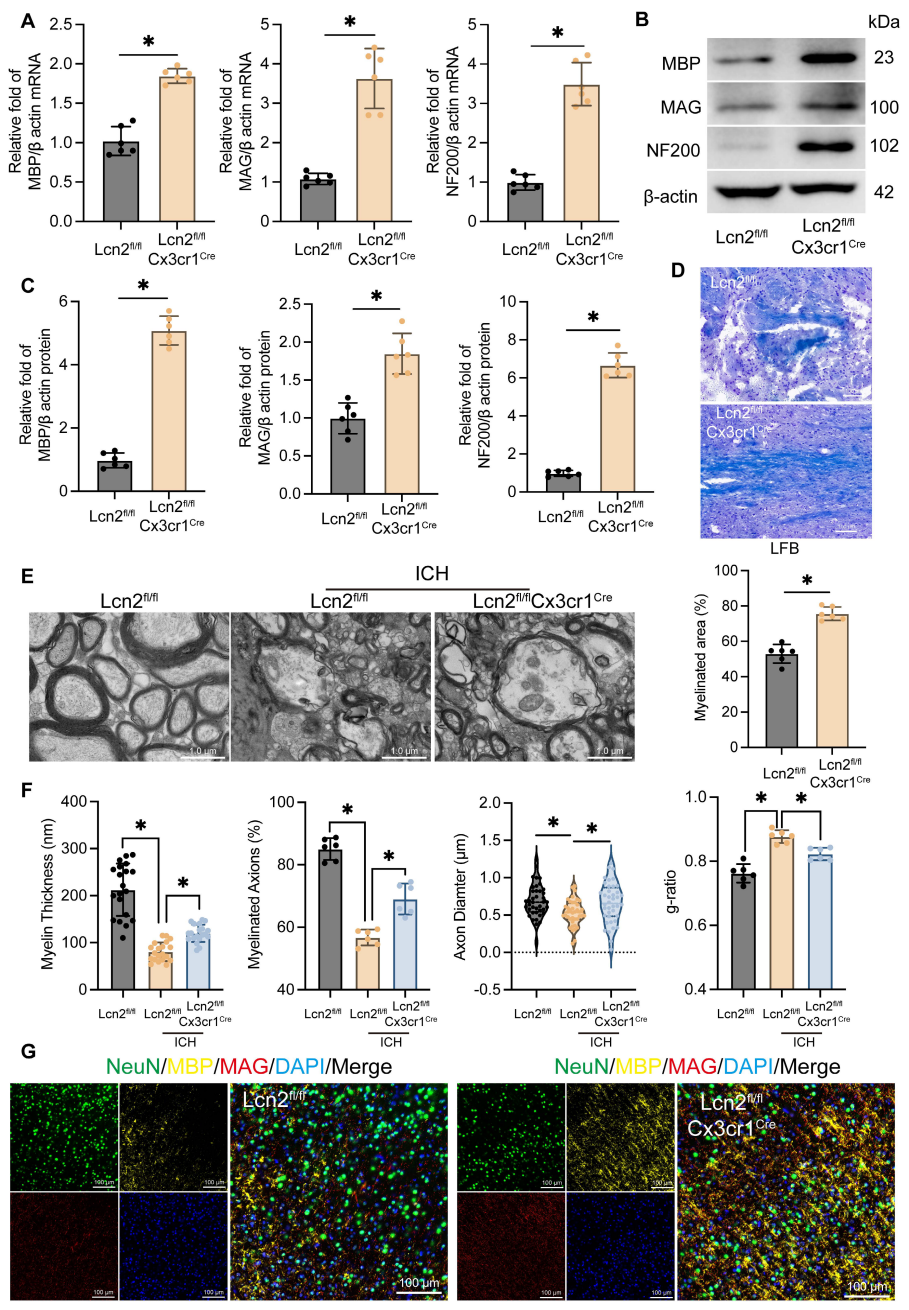
Knockout of Lcn2 by microglia improves myelin sheath recovery in chronic ICH.** A** mRNA levels of MBP, MAG, and NF200 at the site of hemorrhage during the chronic phase of ICH. MBP: t = 9.910, df = 10,* P < 0.0001*. MAG: t = 8.062, df = 10,* P < 0.0001*. NF200: t = 10.57, df = 10,* P < 0.0001*. **B** Protein levels of MBP, MAG, and NF200 at the site of hemorrhage during the chronic phase of ICH.** C** Quantization of results in panel B. MBP: t = 19.66, df = 10,* P < 0.0001*. MAG: t = 6.207, df = 10,* P = 0.0001*. NF200: t = 20.99, df = 10,* P < 0.0001*. **D** LFB staining and quantization of myelinated area. t = 8.538, df = 10,* P < 0.0001*. **E** TEM is used to observe the myelin sheath structure of neurons in each group.** F** Quantization of myelin thickness, myelinated axons, axon diameter, and g-ratio in panel E. myelin thickness: F (2, 57) = 71.42, *P < 0.0001*. myelinated axons: F (2, 15) = 83.76, *P < 0.0001*. axon diameter: F (2, 117) = 5.749, *P = 0.0042*. g-ratio: F (2, 15) = 36.55, *P < 0.0001*. **G** IF is used to detect the co staining of neuronal markers NeuN, MBP, and MAG at the site of hemorrhage during the chronic phase of ICH. The data were analyzed using t-test (**A**, **C** and **D**) or one-way (**F**) analysis of variance and all data are expressed as the mean ± standard deviation. *P < 0.05 represents a statistically significant difference between the two groups. The blots are representative of other replicates in those groups.

Myelin is essential for protecting nerve fibers and facilitating rapid nerve signal transmission, making its effective recovery crucial for improving long-term neurological function in ICH patients 2, 3. To evaluate the impact of Lcn2 knockout in microglia on myelin recovery during the chronic phase of ICH, we analyzed the transcription and translation levels of myelin-associated genes *MBP*, *MAG* and *NF200*. qPCR (Figure 2A) and WB (Figure 2B-C) results showed that, compared to Lcn2^fl/fl^ mice, Lcn2^fl/fl^Cx3cr1^Cre^ mice exhibited significantly higher transcription and translation levels of MBP, MAG, and NF200 at the hemorrhage site during the chronic phase of ICH. LFB staining further demonstrated a larger area of myelin recovery in Lcn2^fl/fl^Cx3cr1^Cre^ mice at the hemorrhage site (Figure 2D). TEM analysis of myelin structure revealed that Lcn2^fl/fl^Cx3cr1^Cre^ mice had superior myelin recovery, as indicated by improved myelin thickness, myelinated axons, axon diameter, and g-ratio (Figure 2E-F). Additionally, triple immunostaining with NeuN, MBP, and MAG confirmed that knocking out Lcn2 in microglia effectively enhances neuronal myelin recovery at the hemorrhage site during the chronic phase of ICH in mice (Figure 2G). In summary, these findings suggest that microglial Lcn2 knockout significantly improves neuronal myelin recovery during the chronic phase of ICH.

### Knocking out Lcn2 facilitates the transformation of microglia to the M2 phenotype and enhances the differentiation of OPCs

**Figure 3:**
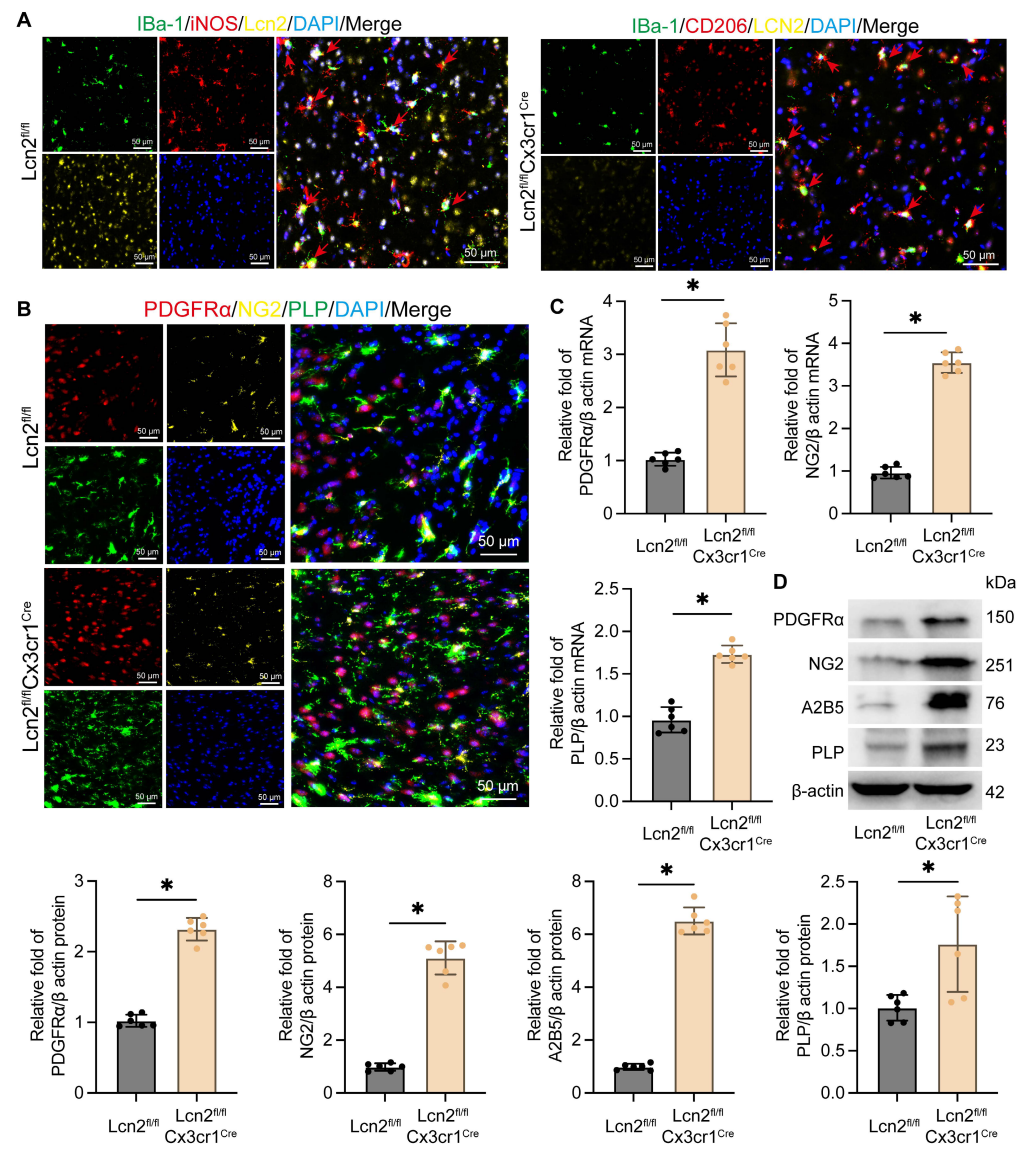
Knockout of Lcn2 promotes phenotypic transformation of microglia and differentiation of OPCs. **A** IF is used to detect the co staining of microglial cell markers iBa-1, iNOS, and Lcn2 at the site of hemorrhage during the chronic phase of ICH. The red arrow represents co stained cells. **B** IF is used to detect the co staining of OPCs markers (PDGFRα and NG2) and oligodendrocyte marker PLP at the site of hemorrhage during the chronic phase of ICH. **C** The mRNA levels of PDGFR α, NG2, and PLP in each group. PDGFR α: t = 9.795, df = 10,* P < 0.0001*. NG2: t = 22.81, df = 10,* P < 0.0001*. PLP: t = 10.39, df = 10,* P < 0.0001*.** D** The protein levels of PDGFR α, NG2, A2B5 and PLP in each group. PDGFR α: t = 17.47, df = 10,* P < 0.0001*. NG2: t = 15.75, df = 10,* P < 0.0001*. A2B5: t = 25.93, df = 10,* P < 0.0001*. PLP: t = 3.144, df = 10,* P = 0.0104*. The data were analyzed using t-test and all data are expressed as the mean ± standard deviation. *P < 0.05 represents a statistically significant difference between the two groups. The blots are representative of other replicates in those groups.

To explore the mechanism by which Lcn2 knockout in microglia enhances myelin recovery in ICH, we first assessed its impact on the microglia themselves. IF results revealed that in Lcn2^fl/fl^ mice, iNOS-positive microglia were predominant, whereas in Lcn2^fl/fl^Cx3cr1^Cre^ mice, there was a higher number of CD206-positive microglia. This indicates that Lcn2 knockout in microglia promotes their transformation to the M2 phenotype (Figure 3A). In addition, previous inflammatory factor measurements in transgenic mice also support that knocking out Lcn2 in microglia can induce a shift toward the M2 phenotype (Figure 1J). Research has shown that following injury, OPCs proliferate and differentiate into mature oligodendrocytes, which are responsible for myelin formation in the CNS. Consequently, we examined specific markers for OPCs (PDGFRα, NG2, and A2B5) and oligodendrocytes (PLP). IF results demonstrated thatLcn2^fl/fl^Cx3cr1^Cre^ ICH mice had a greater number of cells positive for PDGFRα, NG2, and PLP compared to Lcn2^fl/fl^ mice (Figure 3B). Molecular biology assays further indicated that the transcription and translation levels of OPC markers PDGFRα, NG2, and A2B5, as well as the oligodendrocyte marker PLP, were significantly higher in Lcn2^fl/fl^Cx3cr1^Cre^ mice than in Lcn2^fl/fl^ mice (Figure 3C-D). These findings suggest that Lcn2 knockout in microglia facilitates their shift to the M2 phenotype and may enhance the recruitment and differentiation of OPCs into oligodendrocytes, thereby promoting myelin recovery.

### Knocking out Lcn2 in BV2 cells enhances the migration and maturation of OPCs in an *in vitro* co-culture system

**Figure 4:**
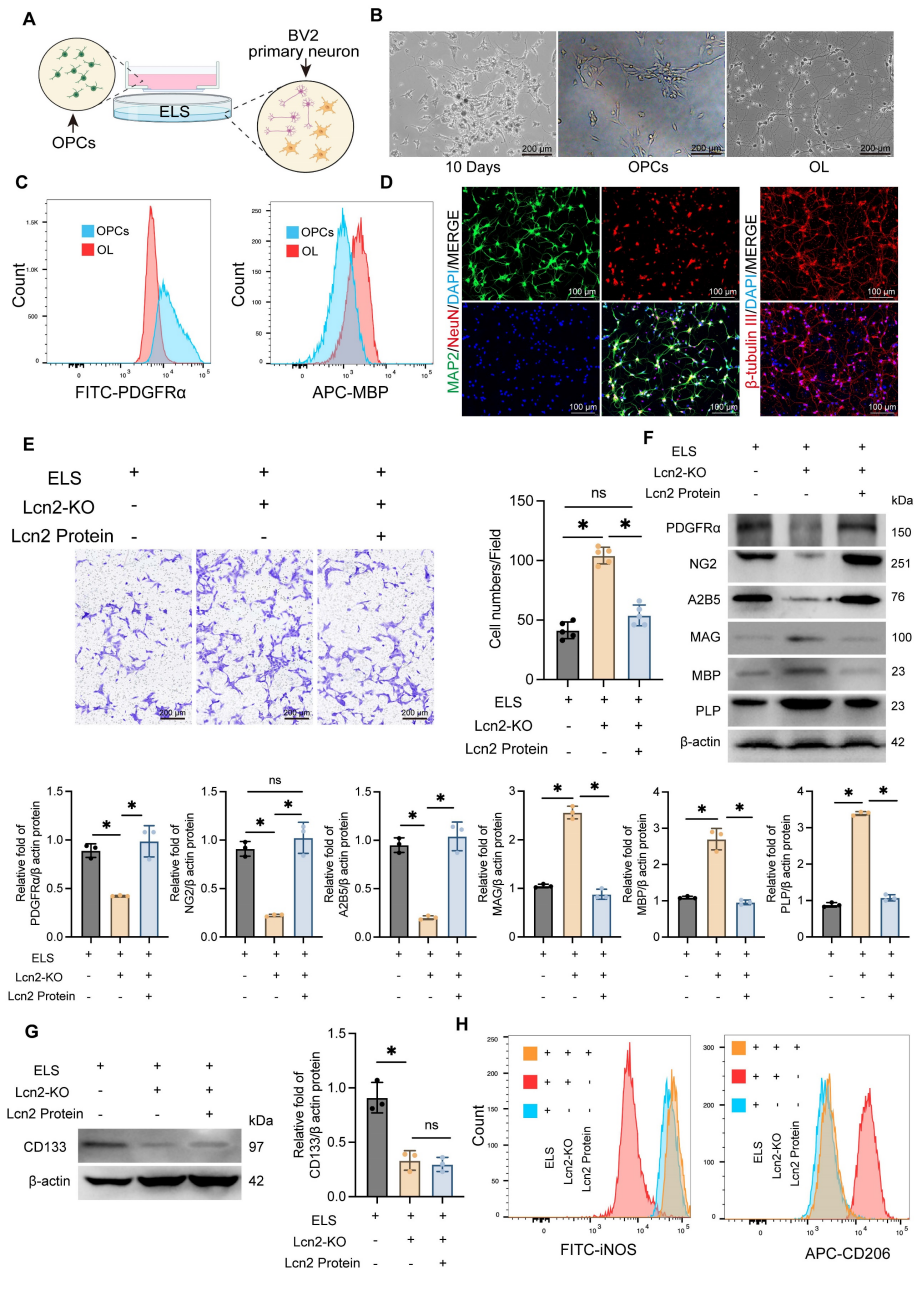
Knockout of Lcn2 in BV2 promotes the migration and differentiation of OPCs *in vitro* co culture system. **A** Schematic diagram of co culture system for *in vitro* experiments.** B** Light microscopy images of extracted OPCs and differentiated oligodendrocytes.** C** Flow cytometry is used to detect the markers PDGFRα for OPCs and MBP for oligodendrocytes.** D** Immunofluorescence detection results of primary neuronal markers MAP2, NeuN, and β - tubulin.** E** Transwell experiment was used to detect the migration ability of OPCs in different groups. F (2, 12) = 95.98, *P < 0.0001*. **F** Protein levels of PDGFRα, NG2, A2B5, MAG, MBP and PLP in OPCs migrating to the lower layer of polyester fiber membrane. PDGFRα: F (2, 6) = 26.29, *P = 0.0011*. NG2: F (2, 6) = 53.26, *P = 0.0002*. A2B5: F (2, 6) = 68.37, *P < 0.0001*. MAG: F (2, 6) = 265.4, *P < 0.0001*. MBP: F (2, 6) = 91.08, *P < 0.0001*. PLP: F (2, 6) = 1254, *P < 0.0001*. **G** Expression level of stemness marker CD133 in OPCs. F (2, 6) = 33.42, *P = 0.0006*.** H** Flow cytometry is used to detect the markers iNOS for M1 BV2 and CD206 for M2 BV2. The data were analyzed using one-way analysis of variance and all data are expressed as the mean ± standard deviation. *P < 0.05 represents a statistically significant difference between the two groups. ns: no statistical difference. The blots are representative of other replicates in those groups.

To further validate the *in vivo* experimental results, we developed a co-culture system using primary neurons, the BV2 cell line, and OPCs (Figure 4A). Initially, OPCs were isolated from cultured tissue using a shaking separation method. By the 10th day of primary culture, two distinct cell morphologies were evident. Most cells were large, adhered closely to the surface, appeared flat with prominent projections, and had large, round nuclei. Another type of cell was observed atop the flat cells, characterized by a smaller size, round or oval shape, strong refractivity, and single or bipolar small projections. At this stage, OPCs were isolated through shaking. The isolated OPCs were then cultured in induction media for an additional 7 days, maturing into oligodendrocytes (Figure 4B). The purity and stemness of the extracted OPCs were confirmed by flow cytometry analysis of PDGFRα and MBP expression levels (Figure 4C, Figure S3A). Primary cortical neurons were identified by immunofluorescence detection of MAP2, NeuN, and β-tubulin expression levels (Figure 4D, Figure S3B).

Using the CRISPR-Cas system, we successfully knocked out Lcn2 in BV2 cells and established a co-culture system with OPCs and primary neurons, incorporating ELS to simulate the ICH environment. We observed that Lcn2 knockout in BV2 cells significantly enhanced OPC migration, an effect that was inhibited by treatment with Lcn2 protein (1μg/mL for 24 h) (Figure 4E). Western blot and qPCR analysis of OPCs that migrated beneath the polyester membrane indicated that Lcn2 knockout in the BV2 culture system significantly promoted the conversion of OPCs to oligodendrocytes, with the Lcn2 protein treatment group showing similar results to the control group (Figure 4F, Figure S3C). Interestingly, while Lcn2 protein inhibited the differentiation of OPCs into oligodendrocytes, the protein and mRNA expression level of CD133 in Lcn2 protein-treated OPCs was lower than in the control group, suggesting that OPCs lost their stemness and differentiation ability following Lcn2 protein treatment (Figure 4G, Figure S3D). Additionally, we assessed phenotypic changes in BV2 cells and primary neuronal activity across different treatment groups. Flow cytometry results showed that Lcn2 knockout in BV2 cells induced a shift from the M1 to M2 phenotype, whereas Lcn2 protein treatment inhibited this transformation (Figure 4H). CCK-8 assay reveals knockout of Lcn2 in BV2 improved neuronal activity in co culture environment, while the group treated with Lcn2 protein had the lowest neuronal activity (Figure S3E). The impact of Lcn2 knockout on the differentiation and stemness of OPCs were separately examined. It was found that in a normal culture environment, the knockout of Lcn2 in OPCs did not affect the protein levels of OPC markers (PDGFRα, NG2, and A2B5), myelin markers (MAG, MBP, and PLP), and the stemness marker CD133 (Figure S4A-B). These findings indicate that, in an *in vitro* co-culture system, Lcn2 knockout in BV2 cells promotes OPC migration and maturation.

### Knocking out Lcn2 may influence the secretion of Gdf-1 in BV2 cells via modulation of the JAK/STAT signaling pathway

**Figure 5:**
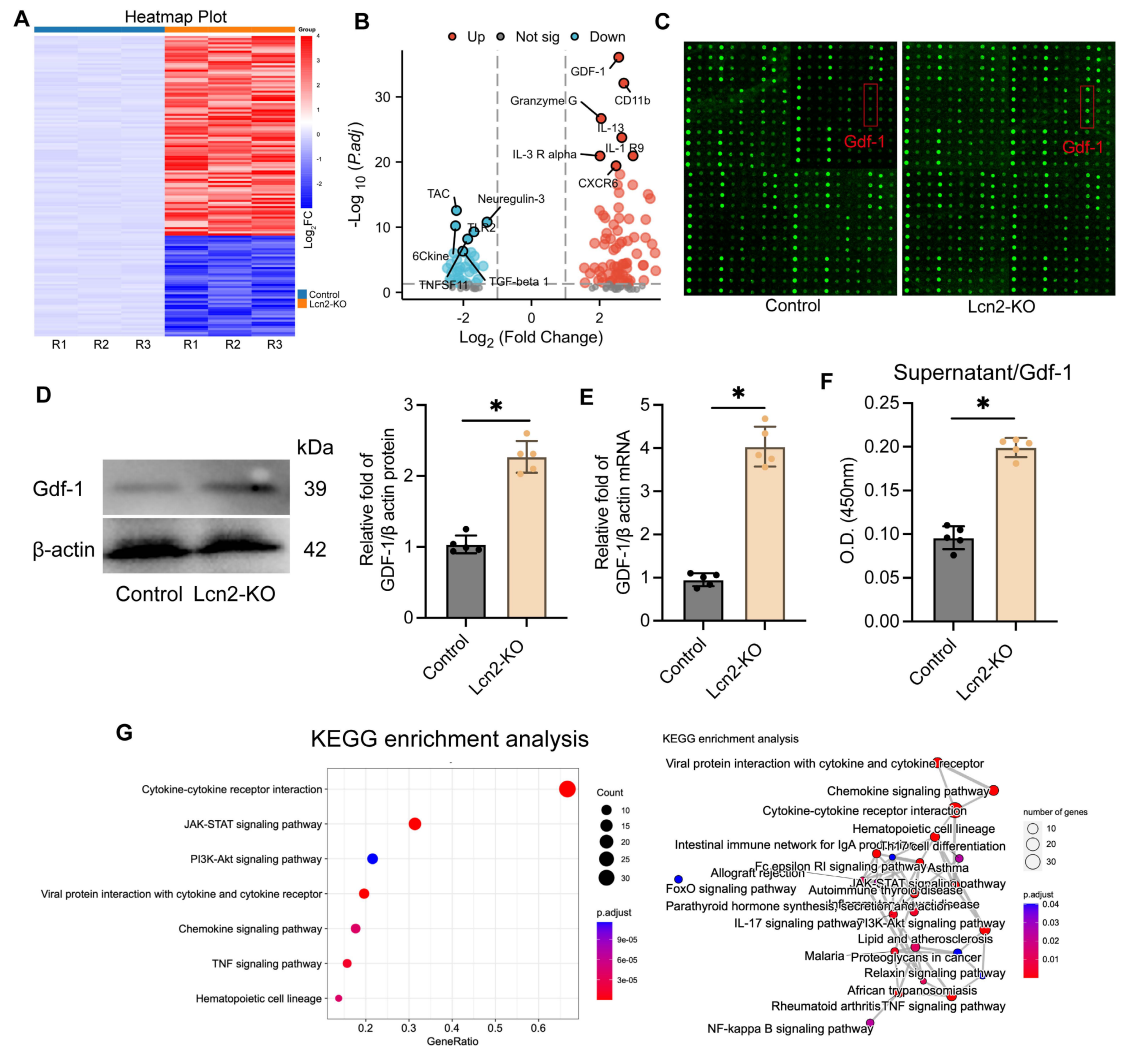
Knocking out Lcn2 may affect the secretion of Gdf-1 in BV2 cells by regulating the JAK/STAT signaling pathway. **A** The heatmap of the results of the mouse cytokine L308 array in BV2 cells and each group consists of three repetitions. **B** Volcanic map display of differentially expressed genes. **C** Fluorescence image of representative blot (Glass carrier). **D** WB detection and quantization of Gdf-1 expression in BV2 cells of each group. t = 10.79, df = 8, *P < 0.0001*. **E** qPCR detection of Gdf-1 mRNA expression in BV2 cells of each group. t = 14.18, df = 8, *P < 0.0001*. **F** ELISA was used to detect the expression level of Gdf-1 in the supernatant of BV2 cells in the co culture system. t = 13.50, df = 8, *P < 0.0001*. **G** KEGG analysis results of differentially expressed genes. The data were analyzed using t-test and all data are expressed as the mean ± standard deviation. *P < 0.05 represents a statistically significant difference between the two groups. The blots are representative of other replicates in those groups.

To further investigate the mechanism of Lcn2 in BV2 cells, we conducted a multi-parameter inflammation chip (The mouse cytokine L308 array) analysis on a co-culture system of normal BV2 cells and Lcn2 knockout BV2 cells (Figure 5A). The results showed a significant upregulation of Gdf-1 expression following Lcn2 knockout (Figure 5B-C). This phenomenon was also confirmed by WB and qPCR analyses (Figure 5D-E). Additionally, ELISA tests on the supernatant samples from the co-culture system indicated a significant increase in Gdf-1 secretion levels after Lcn2 knockout in BV2 cells (Figure 5F). Enrichment analysis of differentially expressed genes between the groups suggested a potential association with the JAK/STAT signaling pathway as indicated by KEGG results (Figure 5G). The GO analysis results suggest that the differentially expressed genes may be related to external side of plasma membrane, receptor ligand activity and cell chemotaxis (Figure S5A-C).

### Jak inhibitor treatment decreased Gdf-1 expression in BV2 cells and inhibited the migration and differentiation of OPCs

**Figure 6:**
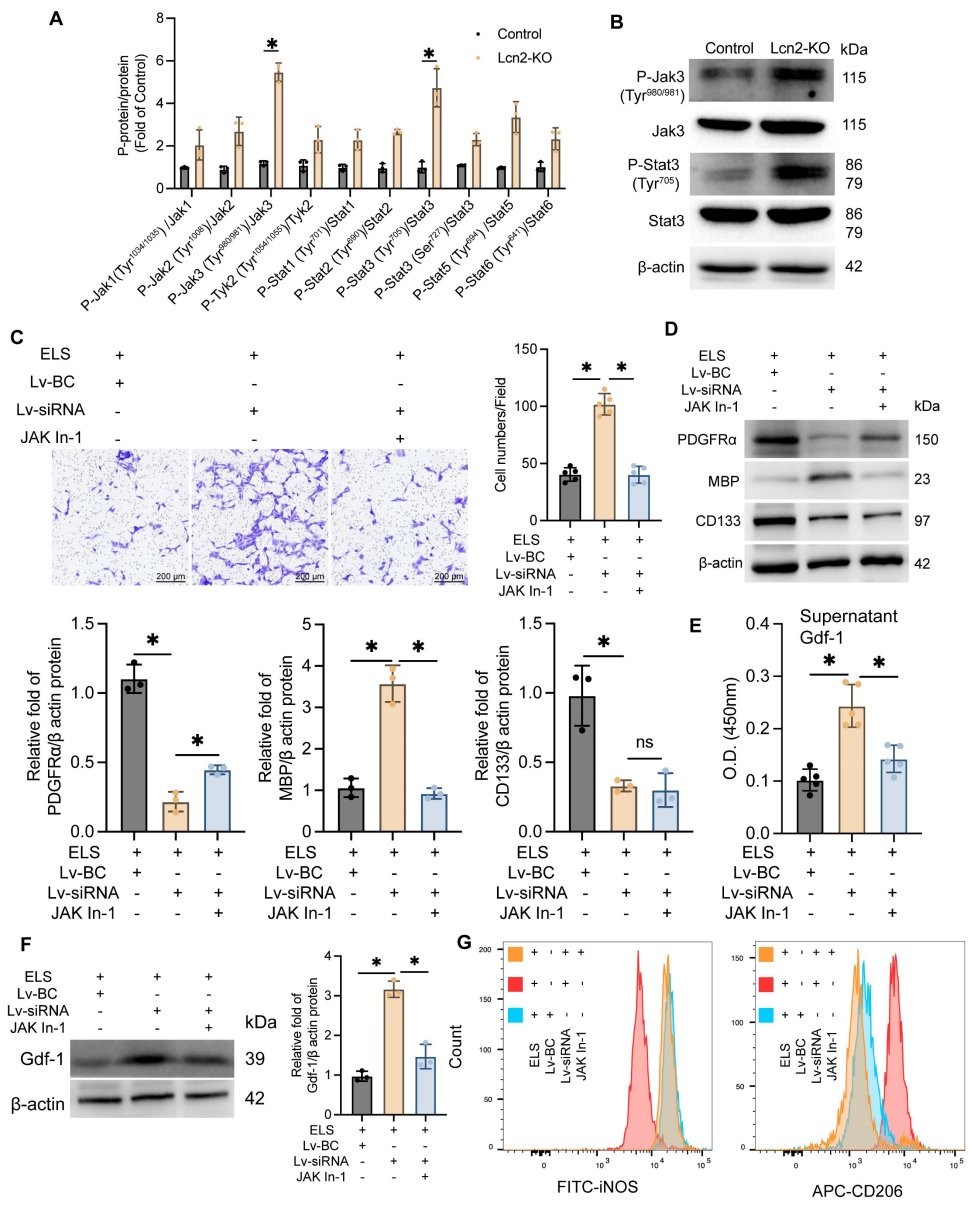
Jak inhibitor treatment enhances Gdf-1 expression in BV2 cells, promoting OPC migration and differentiation. **A** The protein expression levels of Jak and Stat3 family in each group. P-Jak1(Tyr^1034/1035^)/Jak1: t = 2.618, df = 4, *P = 0.0589*. P-Jak2(Tyr^1008^)/Jak2: t = 4.533, df = 4, *P = 0.0106*. P-Jak3(Tyr^980/981^)/Jak3: t = 16.63, df = 4, *P < 0.0001*. P-Tyk2(Tyr^1054/1055^)/Tyk2: t = 3.105, df = 4, *P = 0.0360*. P-Stat1(Tyr^701^)/Stat1: t = 4.497, df = 4, *P = 0.0109*. P-Stat2(Tyr^690^)/Stat2: t = 12.25, df = 4, *P = 0.0003*. P-Stat3(Tyr^705^)/Stat3: t = 6.904, df = 4, *P = 0.0023*. P-Stat3(Ser^727^)/Stat3: t = 7.352, df = 4, *P = 0.0018*. P-Stat5(Tyr^694^) /Stat5: t = 5.660, df = 4, *P = 0.0048*. P-Stat6(Tyr^641^)/Stat6: t = 4.065, df = 4, *P = 0.0153*.** B** The protein expression levels of P-Jak3(Tyr^980/981^), Jak3, P-Stat3(Tyr^705^) and Stat3 in each group.** C** Transwell experiment was used to detect the migration ability of OPCs in different groups. F (2, 12) = 106.1, *P < 0.0001*.** D** The protein expression levels of PDGFRα, MBP and CD133 in each group. PDGFRα: F (2, 6) = 115.5, *P < 0.0001*. MBP: F (2, 6) = 76.37, *P < 0.0001*. CD133: F (2, 6) = 20.99, *P = 0.0020*. **E** ELISA was used to detect the expression level of Gdf-1 in the supernatant of BV2 cells in the co culture system. F (2, 12) = 28.89, *P < 0.0001*.** F** WB detection and quantization of Gdf-1 expression in BV2 cells of each group. F (2, 6) = 77.93, *P < 0.0001*.** G** Flow cytometry is used to detect the markers iNOS for M1 BV2 and CD206 for M2 BV2. The data were analyzed using t-test (**A**) and one-way analysis of variance (**C**, **D**, **E** and **F**) and all data are expressed as the mean ± standard deviation. *P < 0.05 represents a statistically significant difference between the two groups. The blots are representative of other replicates in those groups.

The Jak/Stat family includes several proteins such as JAK1-3, Tyk2, Stat1-3, Stat5, and Stat6. To pinpoint which specific proteins are involved, we assessed the expression levels of all these molecules and their phosphorylated counterparts. Western Blot analysis revealed a significant increase in phosphorylation at the Tyr980/981 site on Jak3 and the Tyr705 site on Stat proteins in Lcn2 knockout mice during the chronic phase of ICH (Figure 6 A-B), indicating that Lcn2 knockout activates the Jak3/Stat3 signaling pathway. In a co-culture system, the addition of 50μM Jak In-1 (a selective Jak3 inhibitor) for 24 h significantly inhibited the enhanced OPC migration induced by Lcn2 interference, as demonstrated by Transwell assays (Figure 6C). Jak In-1 also increased PDGFRα expression and reduced MBP expression in OPCs (Figure 6D). However, Jak In-1 could not restore the decline in OPC stemness caused by Lcn2 knockdown (Figure 6D). Additionally, Jak In-1 treatment suppressed the expression and secretion of Gdf-1 in BV2 cells (Figure 6E-F) and promoted a phenotypic shift in BV2 cells from M2 to M1 (Figure 6G). These findings suggest that in a co-culture system, Lcn2 knockdown enhances BV2 expression and secretion of Gdf-1 protein through activation of the Jak3/Stat3 signaling axis, thereby increasing OPC migration and promoting their maturation into oligodendrocytes. The inhibitor Jak In-1 can reverse the effects induced by Lcn2 knockdown.

### Phosphorylation of Stat3 at the Thr705 site plays a critical role in enhancing the transcription and translation of Gdf-1

**Figure 7:**
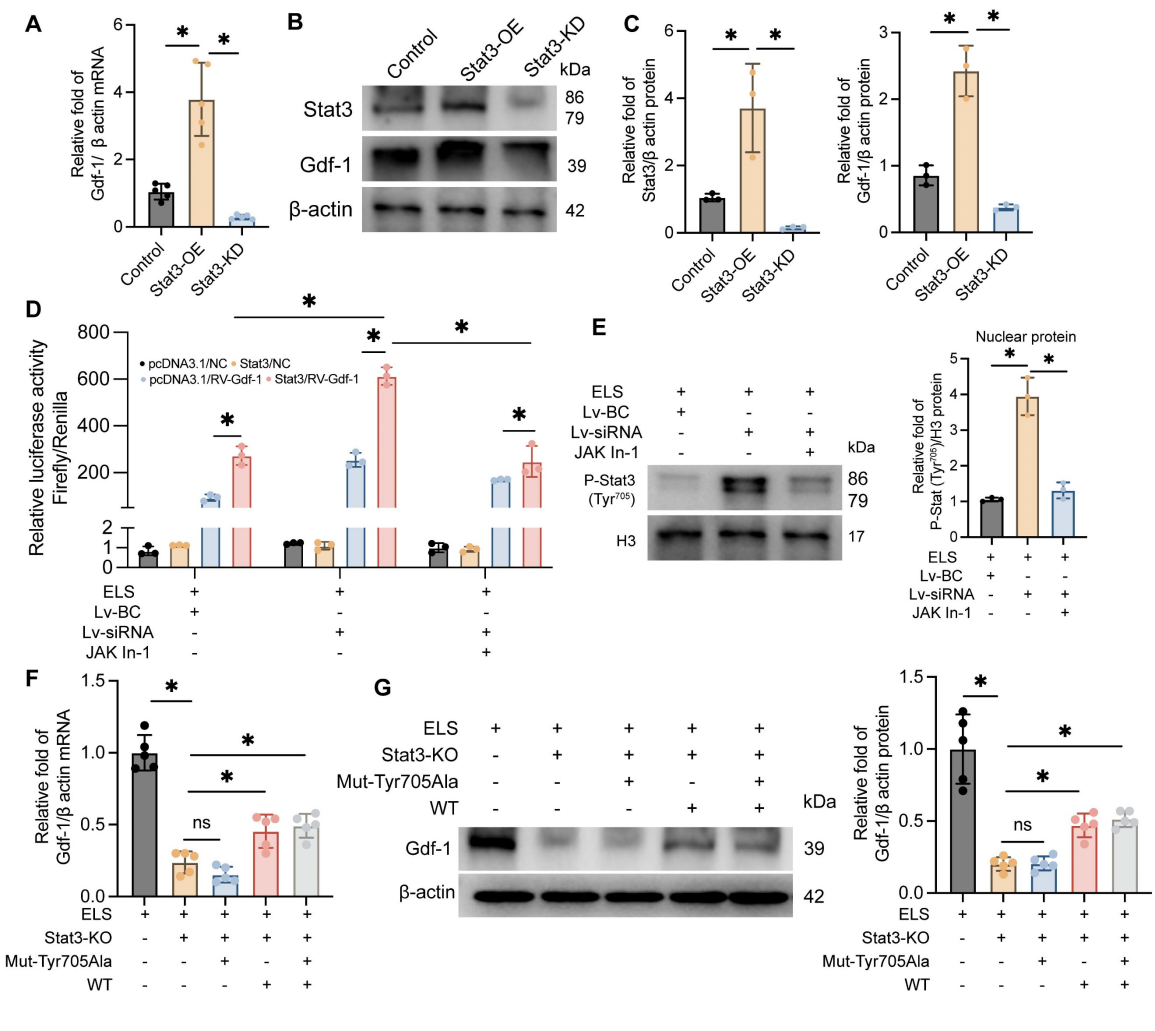
The phosphorylation of Stat3 at Thr705 site is a key factor in regulating Gdf-1 expression. **A** The effect of BV2 differential expression Stat3 on Gdf-1 mRNA levels in *in vitro* ELS environment. F (2, 12) = 40.92, *P < 0.0001*.** B** The effect of BV2 differential expression Stat3 on Gdf-1 protein levels in *in vitro* ELS environment.** C** Quantization of results in panel B. Stat3: F (2, 6) = 17.71, *P = 0.0030*. Gdf-1: F (2, 6) = 60.79, *P = 0.0001*.** D** Dual-luciferase reporter assay was used to detect the regulatory effect of Stat3 on Gdf-1 in BV2 cells of each group. F_Interaction_ (6, 24) = 41.01, *P < 0.0001*. **E** The expression levels of P-Stat3 in the nuclei of each group. F (2, 6) = 69.70, *P < 0.0001*.** F** The effect of Stat3 mutation at amino acid position 705 on Gdf-1 mRNA expression level. F (4, 20) = 61.67, *P < 0.0001*.** G** The effect of Stat3 mutation at amino acid position 705 on Gdf-1 protein expression level. F (4, 20) = 36.61, *P < 0.0001*. The data were analyzed using one-way (**A**, **C, E**, **F** and **G**) or two-way (**D**) analysis of variance and all data are expressed as the mean ± standard deviation. *P < 0.05 represents a statistically significant difference between the two groups. The blots are representative of other replicates in those groups.

Our previous experiments indicate that Gdf-1, synthesized and secreted by BV2 cells, may be a crucial factor in promoting OPCs migration and maturation. As Stat3 is a classical transcription factor, we hypothesized that it might regulate Gdf-1. To test this, we developed BV2 cell lines with Stat3 overexpression and knockdown, adding ELS to the medium to mimic the ICH environment. The molecular biology results demonstrated that Gdf-1 transcription (Figure 7A) and translation levels (Figure 7B-C) were directly proportional to Stat3 levels. Stat3 overexpression significantly boosted Gdf-1 expression, while Stat3 knockdown had the opposite effect. The dual-luciferase reporter assay results indicated that knocking down Lcn2 enhances luciferase expression in the Gdf-1 reporter plasmid, an effect that can be inhibited by Jak In-1 treatment (Figure 7D). Furthermore, Jak In-1 suppressed the nuclear translocation of P-Stat3 induced by Lcn2 knockdown (Figure 7E). To further explore the role of Stat3 phosphorylation at Tyr705 in regulating Gdf-1 expression, the Tyr amino acid at position 705 in the Stat3 sequence was mutated to Ala, resulting in the successful creation of a mutant plasmid. Transfecting the mutant plasmid into Stat3 knockout BV2 cells did not enhance Gdf-1 expression, whereas transfection with the wild-type Stat3 plasmid restored Gdf-1 expression, although differences remained compared to the control group (Figure 7F-G). These findings underscore that phosphorylation of Stat3 at the Thr705 site is a critical factor in enhancing the transcription and translation of Gdf-1.

### The administration of the Lcn2 inhibitor ZINC-94/89 significantly enhanced behavioral performance, mitigated inflammatory responses, and promoted myelin recovery in mice during the chronic phase of ICH

**Figure 8:**
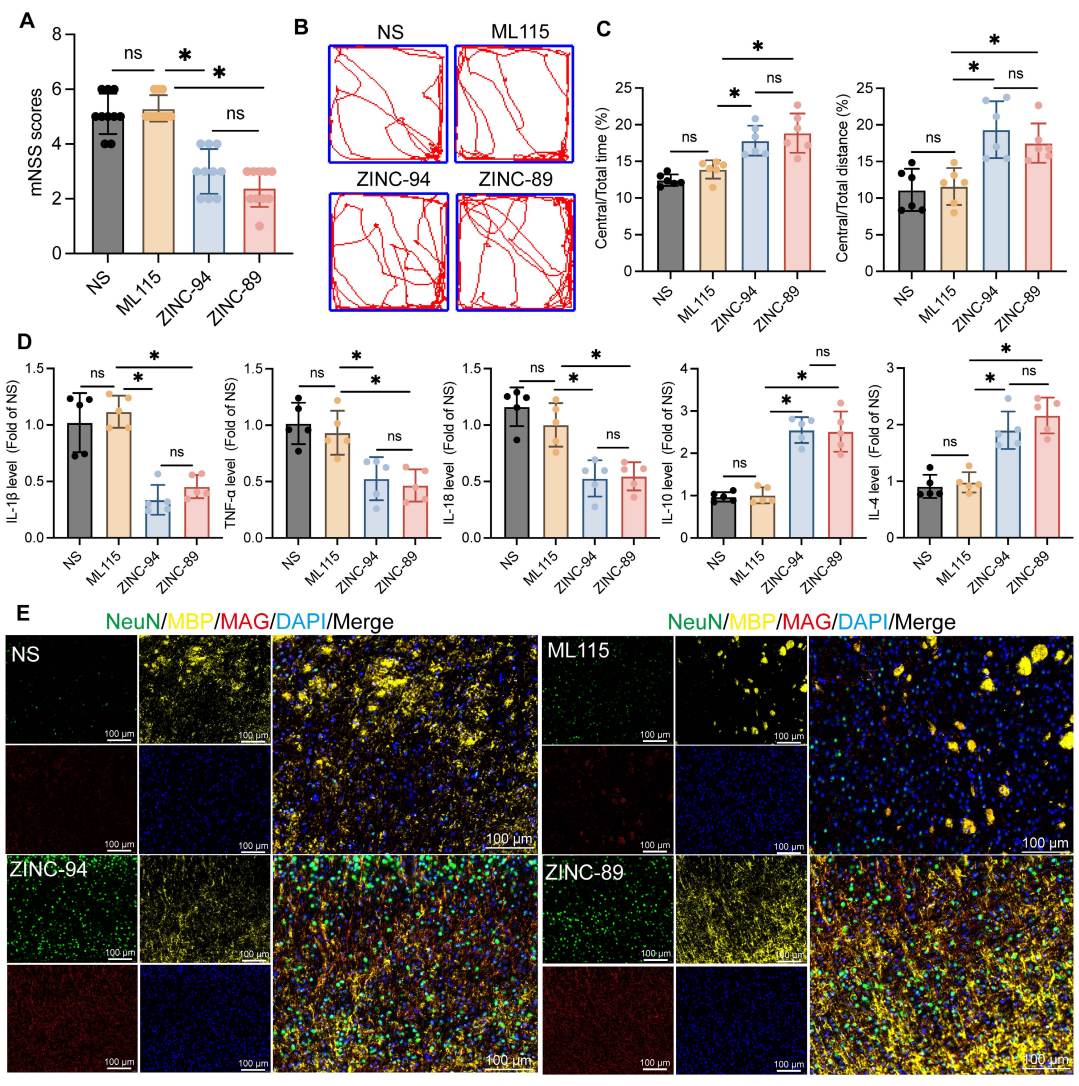
The effects of Lcn2 inhibitor ZINC-94/89 administration on mouse behavior and myelin sheath recovery. **A** mNSS scores of mice in each group. F (3, 36) = 44.48, *P < 0.0001*.** B** Trajectory diagram of mice in open field experiment.** C** Quantization of results in panel B. Central/Total time: F (3, 20) = 16.71, *P < 0.0001*. Central/Total distance: F (3, 20) = 11.21, *P = 0.0002*.** D** ELISA was detected the expression levels of relevant inflammatory factors in each group. IL-1β: F (3, 16) = 26.45, *P < 0.0001*. TNF-α: F (3, 16) = 12.13, *P = 0.0002*. IL-18: F (3, 16) = 19.09, *P < 0.0001*. IL-10: F (3, 16) = 43.17, *P < 0.0001*. IL-4: F (3, 16) = 28.55, *P < 0.0001*. **E** IF is used to detect the co staining of neuronal markers NeuN, MBP, and MAG at the site of hemorrhage during the chronic phase of ICH. The data were analyzed using one-way analysis of variance and all data are expressed as the mean ± standard deviation. *P < 0.05 represents a statistically significant difference between the two groups. ns: no statistical difference.

Previous findings indicate that Lcn2 inhibits the Jak3/Stat3 signaling axis, and knocking out Lcn2 enhances Gdf-1 expression by activating the Jak3/Stat3 pathway, thereby promoting OPCs myelination. Based on this, during the induction of ICH in mice, Lcn2 inhibitors ZINC00784494 (ZINC-94), ZINC00640089 (ZINC-89), and the STAT3 agonist ML115 were administered for three consecutive days at the hemorrhage site to evaluate their effects on recovery during the chronic phase of ICH in mice. Various parameters were assessed three weeks post-treatment. The results showed no significant differences in mNSS scores (Figure 8A) or open field tests (Figure 8B-C) between ICH mice treated with ML115 and NS. However, mice treated with ZINC-89 and ZINC-94 demonstrated significant improvements in behavioral performance compared to the NS group. ELISA results also revealed no significant differences in inflammatory cytokine expression levels between the ML115 and NS groups. In contrast, expressions of inflammatory cytokines IL-1β, TNF-α, and IL-18 were significantly downregulated, while anti-inflammatory factors IL-10 and IL-4 were upregulated in mice treated with ZINC-89 and ZINC-94 during the chronic phase of ICH (Figure 8D). Furthermore, LFB staining analysis (Figure S6A) and assessments of myelin-related molecule expression indicated that myelin recovery was superior in mice treated with ZINC-89 and ZINC-94 compared to the NS and ML115 groups (Figure 8E).

To further investigate the effects of ZINC-89 and ZINC-94 on target cells, IF was used to detect changes in Lcn2 expression across various cell types. The results indicated that the dendritic processes of activated microglia were reduced, and their volume decreased following inhibitor treatment (Figure S7A). The number of neurons also significantly increased after treatment with the inhibitor. Lcn2 was localized in various cell types, but primarily in microglia, and its fluorescence markedly diminished after inhibitor treatment (Figure S7A-C). These findings preliminarily suggest that ZINC-89 and ZINC-94 may exert therapeutic effects by targeting Lcn2 in microglia.

These findings suggest that treatment with Lcn2 inhibitors ZINC-94/89 significantly enhances behavioral performance, reduces inflammatory responses, and improves myelin recovery in mice during the chronic phase of ICH, whereas treatment with the Stat3 activator ML115 shows no clear efficacy in chronic ICH recovery.

## Discussion

**Figure 9:**
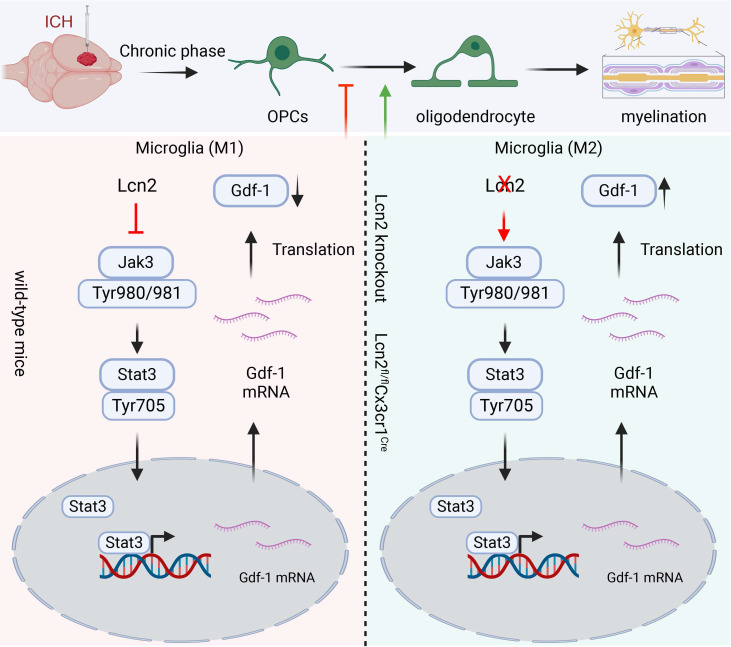
The schematic diagram regarding key parts of this study. Lcn2 knockout promoted microglia transformation to the M2 phenotype and enhanced OPCs differentiation. Mechanistically, Lcn2 knockout might affect Gdf-1 secretion in BV2 cells by modulating the JAK/STAT signaling pathway.

Our study indicated that Knocking out Lcn2 in microglia significantly improved behavioral performance in chronic ICH mice, reduced inflammatory response, and enhanced myelin recovery. Both *in vivo* and *in vitro* experiments confirmed that Lcn2 knockout promoted microglia transformation to the M2 phenotype and enhanced OPCs differentiation. Mechanistically, Lcn2 knockout might affect Gdf-1 secretion in BV2 cells by modulating the JAK/STAT signaling pathway (Figure 9). Treatment with JAK inhibitors decreased Gdf-1 expression in BV2 cells, inhibiting OPCs migration and differentiation. Additionally, phosphorylation of Stat3 at Thr705 plays a critical role in enhancing Gdf-1 transcription and translation. Administration of the Lcn2 inhibitor ZINC-94/89 significantly improved behavioral performance, reduced inflammatory response, and promoted myelin recovery in chronic ICH mice.

ICH not only causes gray matter damage, primarily through the loss of neuronal cell bodies, but also leads to WMI 34. White matter is composed of nerve axons covered by myelin sheaths and oligodendrocytes, which are crucial for protecting neurons and conducting nerve impulses 35. In rodent models of ICH, the death of oligodendrocytes and OPCs can be observed in the affected area as early as the first day after hemorrhagic injury 36, with a noticeable increase in their numbers by the seventh day 37. Mechanistically, WMI following ICH can be categorized into primary and secondary brain injuries 38. Primary brain injury is often due to mechanical damage from the compression of white matter by the hematoma or surrounding edema during the acute phase of ICH. In contrast, secondary brain injury is believed to result from the toxic effects of blood metabolites on the white matter 39. Despite growing attention to WMI after ICH in recent years, effective clinical treatments to rescue WMI and improve neurological deficits post-ICH are still lacking, largely due to limited understanding of the molecular mechanisms involved 39, 40. Although previous studies have shown that rodent models of ICH can accurately replicate the natural progression of human ICH, there is still a lack of clinical trial evidence to confirm similar phenomena in the brains of ICH patients 37, 41. While WMI damage occurs during the acute phase of ICH, clinical focus is often on removing cerebral hematomas, preventing cerebral edema, and prioritizing survival in severe cases. However, chronic WMI damage is one of the most significant factors affecting the quality of life for patients, which is why our research is more focused on myelin recovery and potential mechanisms during the chronic phase of ICH in mice.

In rodent and large animal models like pigs, demyelination, axonal damage, and oligodendrocytes death are the main pathological changes associated with WMI following ICH 40, 42. Besides the primary WMI that occurs in the lesion area, distal axons from the lesion also undergo degeneration and degradation, such as widespread Wallerian degeneration occurring in the distal corticospinal tract after ICH. However, apart from the involvement of the NOD-like receptor family pyrin domain-containing 3 inflammasome, other mechanisms remain unclear 43. In the lesion area, WMI is mostly observed in the periphery of the hematoma and corpus callosum, with the anterior commissure being less affected 44. Studies report that on the first day after ICH, immunohistochemical staining with MBP and dMBP reveals partial axonal loss and fragmentation of the myelin sheath in the central and peripheral areas of the hematoma 45. This pathological change peaks on the third day after ICH, showing disrupted axonal size and shape, reduced axon numbers, degradation of myelin basic protein within the myelin sheath, and swollen and degenerating neurofilaments 42. By the 28th day post-ICH, the extent of WMI decreases but does not return to baseline 46, aligning with our research findings. Interestingly, studies report no significant difference in the timing or extent of demyelination between young and old rats, yet older rats experience more severe and prolonged axonal damage, which may explain the slower neural recovery observed in older rats after ICH 45.

Normal myelin is typically marked by MBP, while dMBP serves as a marker for demyelination. Increased expression of amyloid precursor protein or neurofilament heavy polypeptide can indicate axonal injury. LFB is used to identify normal myelin in lesion areas and is commonly employed to detect late-stage WMI after ICH 46, 47. Additionally, oligodendrocyte lineage cell death is observed during WMI, which can be labeled with dyes like TUNEL and PI 36. The oligodendrocyte lineage includes mature oligodendrocytes that secrete myelin, OPCs, and immature oligodendrocytes 41. Oligodendrocyte transcription factor1/2 (Olig1/2), Sox10, and Nkx2.2 are present throughout oligodendrocyte development, serving as markers for the oligodendrocyte lineage without distinguishing different stages 41. Researchers often use Olig2 and NG2 to double-label OPCs, while Olig2 combined with adenomatous polyposis coli expressed in mature oligodendrocytes, marks mature oligodendrocytes 37. Interestingly, some studies report A2B5 as a marker for OPCs, detectable only in rats, but our research also identified A2B5 in mouse OPCs. It's important to note that NG2 is also expressed in pericytes surrounding capillaries and venous endothelial cells 48. Transmission electron microscopy can examine the number, morphology, axonal gaps, and myelin thickness of myelinated axons in experimental animals 49. However, in clinical settings, the difficulty in obtaining pathological slices, the lengthy preparation time, and the invasive nature of these procedures make immunohistochemistry or transmission electron microscopy rare for detecting WMI after ICH. In contrast, non-invasive imaging techniques like magnetic resonance imaging (MRI) are often used to construct3D images of brain tissue to assess white matter structure and function 50.

After ICH in mice, there is a compensatory increase in OPCs in the lesion area, but the mechanism behind this increase—whether it involves proliferation or migration—is still unclear. Due to the limitations of *in vivo* experiments in dynamically tracking the progression of OPCs, it is difficult to determine their source. However, in cell experiments, we observed that OPCs can migrate to the lower chamber of Transwell plates, and Lcn2 knockout reduced the stemness of OPCs, promoting their differentiation into oligodendrocytes. The proliferation of OPCs depends on their stemness level. Therefore, we speculate that the increased OPCs in the ICH region are likely derived from migration rather than proliferation. Furthermore, these phenomena have sparked interest in using drugs to promote OPC differentiation 51. Studies have shown that insulin-like growth factor-1 and platelet-derived growth factor can encourage OPC differentiation *in vitro* 52, 53. In a rat ICH model using autologous blood injection, Yang and colleagues used NG2 and CNPase to label OPCs and mature oligodendrocytes, respectively. They found that a 12 mg/kg dose of thymosin β4 significantly boosted the proliferation and differentiation of OPCs around the hematoma compared to a saline-injected control group. This led to the formation of mature oligodendrocytes, repairing damaged myelin and improving neurological function in rats. Moreover, drugs like the insulin sensitizer rosiglitazone, the antihistamine clemastine, and vitamin D have also been shown to promote OPC proliferation and differentiation. However, these effects have been observed in mouse models of WMI-related diseases (such as ischemic stroke, multiple sclerosis, and spinal cord injury) and still need to be validated in ICH models 54, 55. Our research found that Lcn2 expression in microglia interferes with their analysis of the Gdf-1 cytokine, and an *in vitro* co-culture system confirmed that Gdf-1 effectively promotes OPC migration and maturation into oligodendrocytes. However, although our research findings provide new insights and methods for modulating OPCs differentiation, we did not construct transgenic mice with specific deletion of Lcn2 in oligodendrocytes for this study due to that the OPC-specific knockout mice available on the market primarily originate from the tamoxifen-induced NG2-CreERT mice developed by Jackson Laboratory, rather than from the conventional Flox-Cre system. Therefore, we intervened in Lcn2 in OPCs using AAVs and found that Lcn2 knockout in OPCs had a lesser therapeutic effect on mice after ICH compared to Lcn2 knockout in microglia. Results from cellular experiments also indicated that Lcn2 knockout in OPCs does not affect their differentiation and stemness. This may be because Lcn2 itself is a member of the inflammatory family rather than a structural component of myelination. Thus, the impact of Lcn2 on myelination is more attributed to its regulation of microglial responses to inflammation rather than a direct modulation of OPCs function. Additionally, this study primarily focused on the chronic phase of ICH, while the role of Lcn2 during the acute phase remains unclear. Although we offer a new perspective, the potential mechanisms by which Lcn2 knockout affects OPC differentiation and myelin repair are not fully understood and require further investigation to elucidate the detailed molecular pathways involved.

In our study, we identified several potential targets for the treatment of chronic phase ICH-related white matter recovery. Although our findings suggest that these interventions may have ideal efficacy, there are still some limitations. For instance, the results from the inflammatory factor chip indicate that Gdf-1 may promote the differentiation of OPCs and the recovery of myelin. However, literature reports suggest that the inflammatory balance itself plays a regulatory role in the damage and recovery of myelin. Additionally, Lcn2 inhibitors have also significantly regulated the secretion of inflammatory factors and promoted myelin recovery. Therefore, the efficacy of white matter recovery in the chronic phase of ICH cannot be entirely attributed to the function of Gdf-1. It is possible that the secretion of Gdf-1 only provides a limited impact. Furthermore, although the Lcn2 inhibitor Zn-94/89 has shown efficacy, there is a lack of exploration regarding its maximum efficacy and timing, necessitating further research into pharmacokinetics, pharmacodynamics, or dose-gradient experiments. It remains unclear whether the efficacy of the inhibitor Zn-94/89 is due to the inhibition of Lcn2 in microglia and whether it has a broader target cell mediation. Finally, the overexpression of Stat3 increased the expression of Gdf-1, while the Stat3 agonist ML115 appeared to have no therapeutic effect. This may also be attributed to the multifunctionality and multidimensionality of the Jak/Stat signaling axis in molecular signaling during disease. A single regulation of Stat3, located at the traffic hub of the signaling axis, may struggle to effectively target the improvement of disease efficacy.

On the other hand, to better simulate the complex multicellular environment *in vivo* and consider the impact of cell interactions on the results, we co-cultured two types of cells, primary neurons and BV2 cells, in the lower chamber of the co-culture system. Unfortunately, we did not use primary microglia for the experiments. There are three considerations for this decision: First, the extraction processes for primary microglia and primary neurons are completely different. The two types of primary cells need to be cultured in different environments for selection. To co-culture these two primary cell types, one of the primary cells would need to undergo trypsin digestion and be replanted, which could significantly affect the state of the primary cells and interfere with the experimental results. Second, the culture systems for primary microglia and primary neurons differ, making it difficult to control the state of both cell types when mixed together, especially for microglia. Third, our study involves using CRISPR/Cas9 technology to knock out the Lcn2 gene in microglia. This process requires long-term selection, and primary microglia cannot be proliferated and cultured for extended periods. These reasons are also why we ultimately chose to use the BV2 cell line for co-culture.

ICH is both a hot topic and a challenging area in CNS disease research. Traditionally, research has focused on neuron repair and regeneration, given that neurons are seen as the fundamental functional unit of brain tissue, but this has had limited clinical impact on ICH treatment 56. Researchers are increasingly recognizing the potential of targeting the protection and regeneration of oligodendrocytes, given the role of white matter in neuroprotection and the conduction of impulses across brain regions, which may aid in treating ICH. Clinically, there's a lack of large-scale statistical data on WMI after ICH, both domestically and internationally. Basic information like its incidence and correlation with prognosis is still scarce. However, numerous clinical cases indicate that WMI is quite common in ICH patients and is a significant feature of the condition 57. Extensive animal studies have shown that WMI after ICH can lead to emotional, cognitive, and sensorimotor impairments in animals, and saving damaged white matter can alleviate these symptoms 38, 58. Since human white matter accounts for 50% of brain volume—higher than that in experimental animals—there is reason to believe that WMI post-ICH could have more severe impacts on patients. Over the past 20 years, significant progress has been made in understanding the molecular mechanisms of WMI after ICH, including the mass effect of brain hematomas and surrounding edema, toxicity from various biochemical metabolites, glutamate-mediated neurotoxicity, and neuroinflammatory responses. Although various interventions targeting these mechanisms have been proposed, it remains uncertain which are applicable for clinical treatment. Current research on WMI after ICH still faces several challenges: first, the precision and developmental differences between human embryos and the animals like mice, rats, pigs, and rabbits mean these pathophysiological mechanisms might not fully apply to humans; second, these treatments have been developed in animal models and do not yet account for the complexity seen in clinical patients; third, most treatments focus on singular interventions, while multi-target, multi-modal, and multi-strategy combination treatments are still in the design and testing stages; finally, WMI is also a complication of other CNS diseases such as ischemic stroke, multiple sclerosis, and traumatic brain injury, necessitating further research on the distinctions and commonalities of WMI in these conditions. Further exploration of the pathophysiological mechanisms and therapeutic strategies for WMI after ICH is needed to advance understanding and clinical treatment of the condition.

In conclusion, our study showed that knocking out Lcn2 in microglia significantly improved behavioral performance in chronic ICH mice, reduced inflammatory response, and enhanced myelin recovery. Both *in vivo* and *in vitro* experiments confirmed that Lcn2 knockout promoted microglia transformation to the M2 phenotype and enhanced OPCs differentiation. Administration of the Lcn2 inhibitor ZINC-94/89 significantly improved behavioral performance, reduced inflammatory response, and promoted myelin recovery in chronic ICH mice.

## Supplementary Material

Supplementary figures and table.

## Abbreviations

ICH: Intracerebral Hemorrhage
OPCs: Oligodendrocyte Precursor Cells
CNS: Central Nervous System
Lcn2: Lipocalin-2
Gdf-1: Growth differentiation factor 1
PCR: Polymerase Chain Reaction
MWM: Morris Water Maze
ELISA: Enzyme-Linked Immunosorbent Assay
qPCR: quantitative Polymerase Chain Reaction
IF: Immunofluorescence
WB: Bestern Blot
TEM: Transmission Electron Microscopy
LFB: Luxol Fast Blue
mNSS: modified Neurological Severity Score
ZINC-89: ZINC00640089
ZINC-94: ZINC00784494
WMI: White Matter Injury
MRI: Magnetic Resonance Imaging
DWI: Diffusion-Weighted Imaging
DTI: Diffusion Tensor Imaging
FZKS: Fazekas scale
FA: Fractional Anisotropy
RD: Radial Diffusivity
AD: Axial Diffusivity
