# Supplementary material for: Microglial Lcn2 knockout enhances chronic intracerebral hemorrhage recovery by restoring myelin and reducing inflammation
Source: Theranostics. 2025 Mar 29;15(10):4763–84. doi: 10.7150/thno.109440 (PMC11984404; doi:10.7150/thno.109440)
Supplement: Supplementary file 1 — Supplementary figures and table. [file thnov15p4763s1.pdf]

Table S1: Primer information for qPCR experiment

Gene	Primer Information
MBP(Mus)-F	5’-AATCGGCTCACAAGGGATTCA-3’
MBP(Mus)-R	5’-TCCTCCCAGCTTAAAGATTTTGG-3’
MAG(Mus)-F	5’-CTGCCGCTGTTTTGGATAATGA-3’
MAG(Mus)-R	5’-CATCGGGGAAGTCGAAACGG-3’
NF200(Mus)-F	5’-AGACCCCCGTCAAGGAAGG-3’
NF200(Mus)-R	5’-CTTCTCAGGGGATTTCGCCT-3’
PDGFRα(Mus)-F	5’-AGAGTTACACGTTTGAGCTGTC-3’
PDGFRα(Mus)-R	5’-GTCCCTCCACGGTACTCCT-3’
NG2(Mus)-F	5’-GGGCTGTGCTGTCTGTTGA-3’
NG2(Mus)-R	5’-TGATTCCCTTCAGGTAAGGCA-3’
PLP(Mus)-F	5’-CCAGAATGTATGGTGTTCTCCC-3’
PLP(Mus)-R	5’-GGCCCATGAGTTTAAGGACG-3’
Gdf-1(Mus)-F	5’-AACTAGGGGTCGCCGGAAA-3’
Gdf-1(Mus)-R	5’-TCAAAGACGACTGTCCACTCG-3’

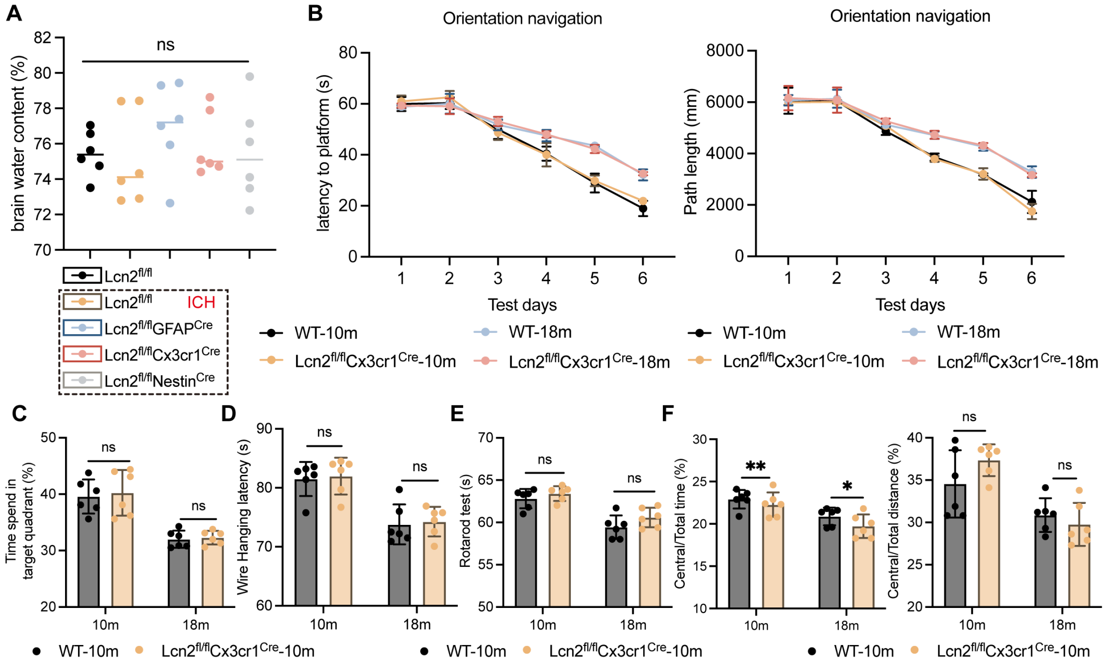

Figure S1. brain water content in each group and the long-term effects of Lcn2 knockout on brain function.

A Detection of brain water content in each group. F (4, 25) = 0.5906, *P = 0.6726*. **B** Quantification of orientation navigation results in each group. **C** Quantization of the probe test results in each group $F_{interaction}(1, 20) = 0.02750$, *P = 0.8700*. **D** Quantify the latency of the Wire Hanging experiment $F_{interaction}(1, 20) = 4.636e\text{-}005$, *P = 0.9946*. **E** Quantify of rotarod test $F_{interaction}(1, 20) = 0.3409$, *P = 0.5658*. **F** quantitative results of open field experiment in each group. Central/Total time: $F_{interaction}(1, 20) = 0.2528$, *P = 0.6206*; Central/Total distance: $F_{interaction}(1, 20) = 3.071$, *P = 0.0950*. The data were analyzed using one-way (A) or two-way (B-F) analysis of variance and all data are expressed as the mean ± standard deviation. ns: no statistical difference.

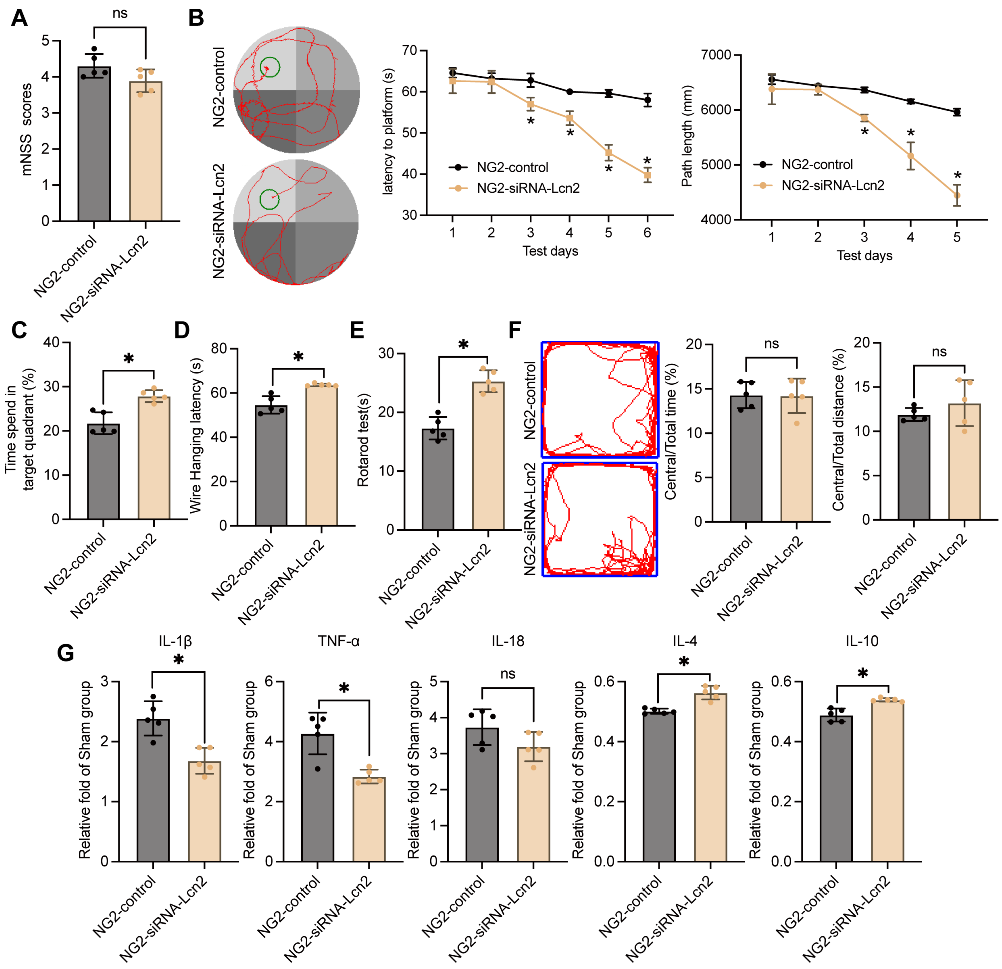

Figure S2. The impact of knocking out Lcn2 in OPCs using AAVs on myelin recovery in ICH mice.

A mNSS scores: t = 2.047, df = 8, $P = 0.0749$. **B** Representative trajectory diagram of the sixth day of orientation navigation phase and quantization in the MWM. Latency: $F_{Interaction}$ (5, 48) = 38.13, $P < 0.0001$. Path length: $F_{Interaction}$ (4, 40) = 43.79, $P < 0.0001$. **C** Quantization of result in the probe test in MMW t = 4.866, df = 8, $P = 0.0012$. **D** Quantify the latency of the Wire Hanging experiment t = 5.056, df = 8, $P = 0.0010$. **E** Quantify of rotarod test t = 6.678, df = 8, $P = 0.0002$. **F** Trajectory diagram and quantitative results of mice in open field experiment. Central/Total time: t = 0.07323, df = 8, $P = 0.9434$. Central/Total distance: t = 1.078, df = 8, $P = 0.3123$. **G** ELISA was detected the expression levels of relevant inflammatory factors. IL-1β: t = 4.421, df = 8, $P = 0.0022$. TNF-α: t = 4.385, df = 8, $P = 0.0023$. IL-18: t = 1.885, df = 8, $P = 0.0961$. IL-4: t = 5.679, df = 8, $P = 0.0005$. IL-10: t = 5.039, df = 8, $P = 0.0010$. The data were analyzed using student T test (**A**, **C, D**, **E**, **F** and **G**) or two-way (**B**) analysis of variance and all data are expressed as the mean ± standard deviation. *$P < 0.05$ represents a statistically significant difference between the two groups. ns: no statistical difference.

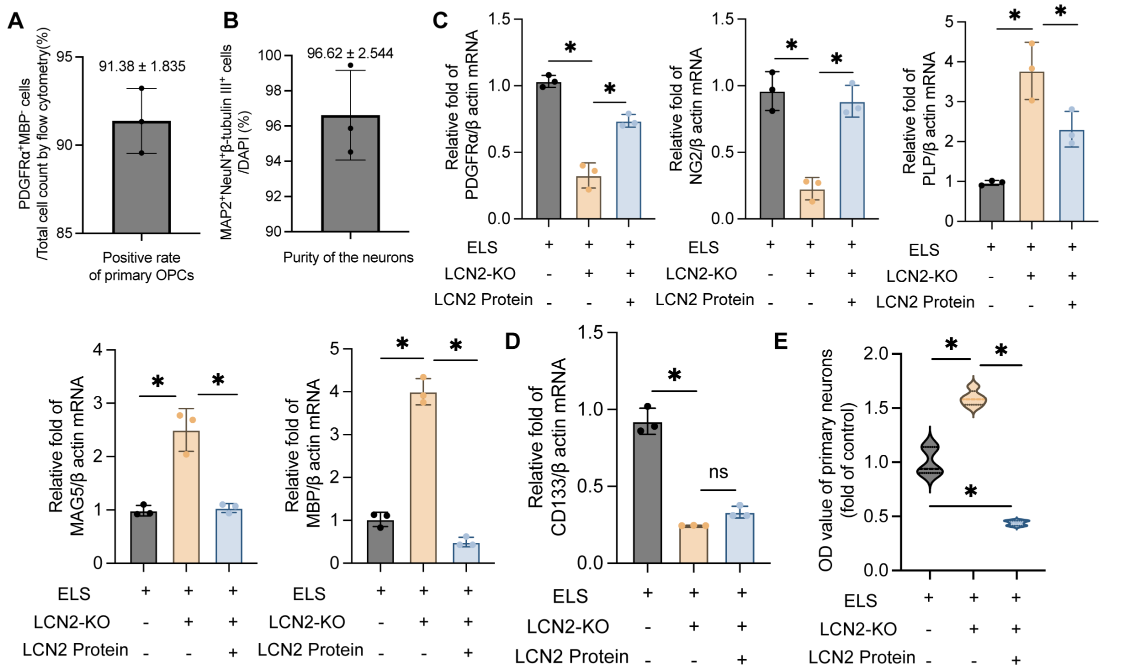

Figure S3. Knockout of Lcn2 in BV2 promotes the migration and differentiation of OPCs in vitro co culture system.

A Positive rate of primary OPCs (91.38 ± 1.835). **B** Purity of Primary Neurons (96.62 ± 2.544). **C** mRNA levels of PDGFRα, NG2, MAG, MBP and PLP in OPCs migrating to the lower layer of polyester fiber membrane. PDGFRα: F (2, 6) = 85.85, $P < 0.0001$. NG2: F (2, 6) = 34.52, $P = 0.0005$. MAG: F (2, 6) = 37.52, $P = 0.0004$. MBP: F (2, 6) = 241.2, $P < 0.0001$. PLP: F (2, 6) = 24.81, $P = 0.0013$. **D** Expression mRNA level of stemness marker CD133 in OPCs. F (2, 6) = 140.6, $P < 0.0001$. E CCK-8 assay is used to detect neuronal activity. F (2, 12) = 230.4, $P < 0.0001$. The data were analyzed using one-way analysis of variance and all data are expressed as the mean ± standard deviation. $^*P < 0.05$ represents a statistically significant difference between the two groups.

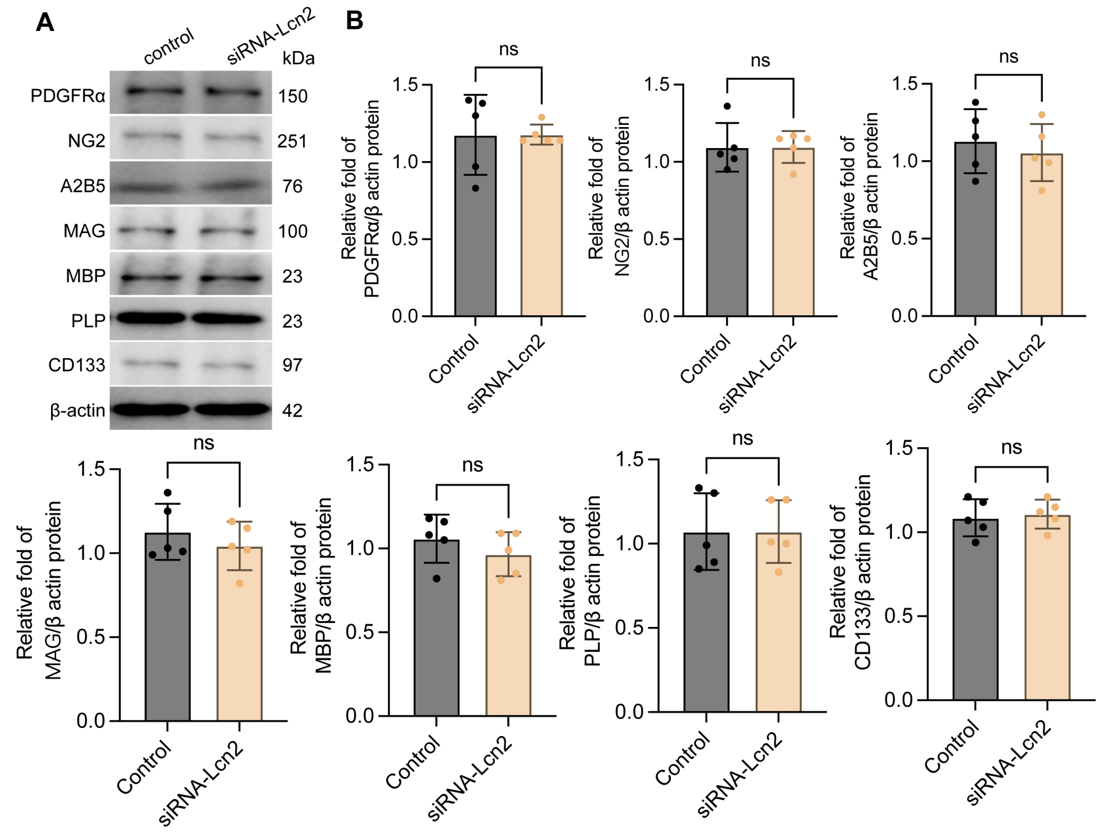

Figure S4. The impact of Lcn2 knockout on the differentiation and stemness of OPCs.

A The protein levels of OPC markers (PDGFRα, NG2, and A2B5), myelin markers (MAG, MBP, and PLP), and the stemness marker CD133 were detected by WB. **B** Quantization of result in panel A. PDGFRα: t = 0.01672, df = 8, $P = 0.9871$. NG2: t = 0.02380, df = 8, $P = 0.9816$. A2B5: t = 0.5974, df = 8, P = 0.5668. MAG: t = 0.8516, df = 8, $P = 0.4192$. MBP: t = 1.057, df = 8, $P = 0.3215$. PLP: t = 0.000, df = 8, $P > 0.9999$. CD133: t = 0.3514, df = 8, $P = 0.7344$. The data were analyzed using student T test and all data are expressed as the mean ± standard deviation. *$P < 0.05$ represents a statistically significant difference between the two groups.

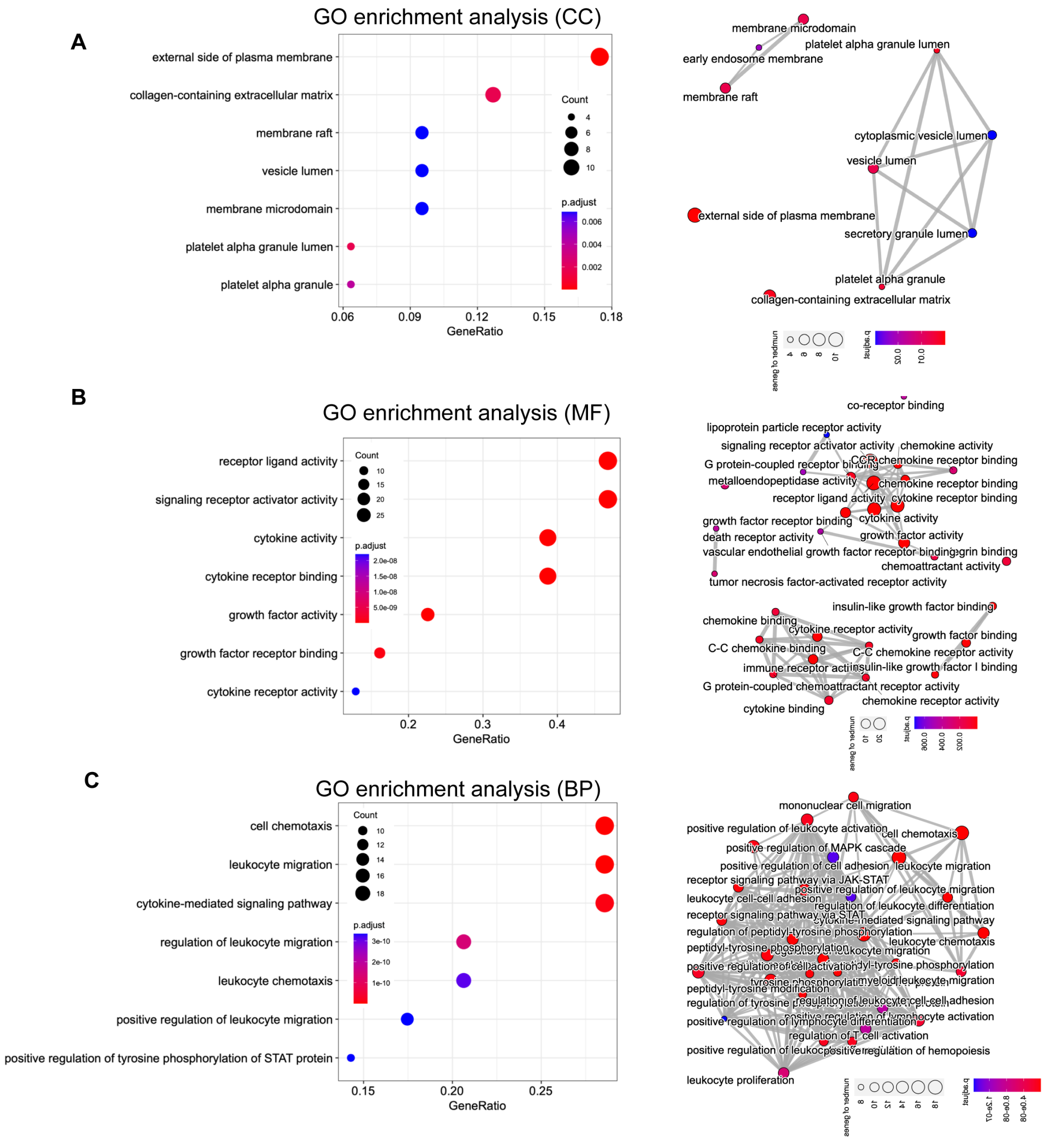

Figure S5. GO analysis results of differentially expressed genes.

A Cellular component (CC) of GO analysis. **B** Molecular function (MF) of GO analysis. **C** Biological process (BP) of GO analysis.

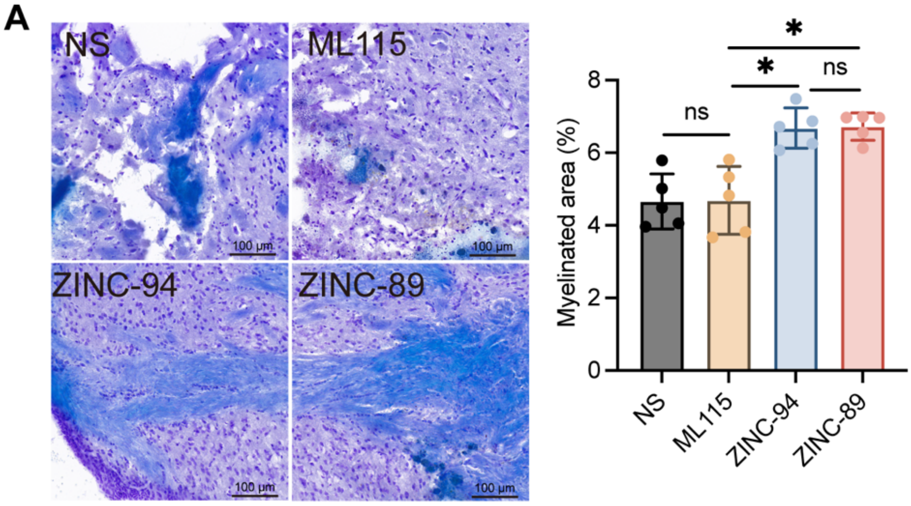

Figure S6. LFB staining.

A LFB staining analysis and quantization of result. F (3, 16) = 14.44, $P < 0.0001$. The data were analyzed using one-way analysis of variance and all data are expressed as the mean ± standard deviation. $^*P < 0.05$ represents a statistically significant difference between the two groups. ns: no statistical difference.

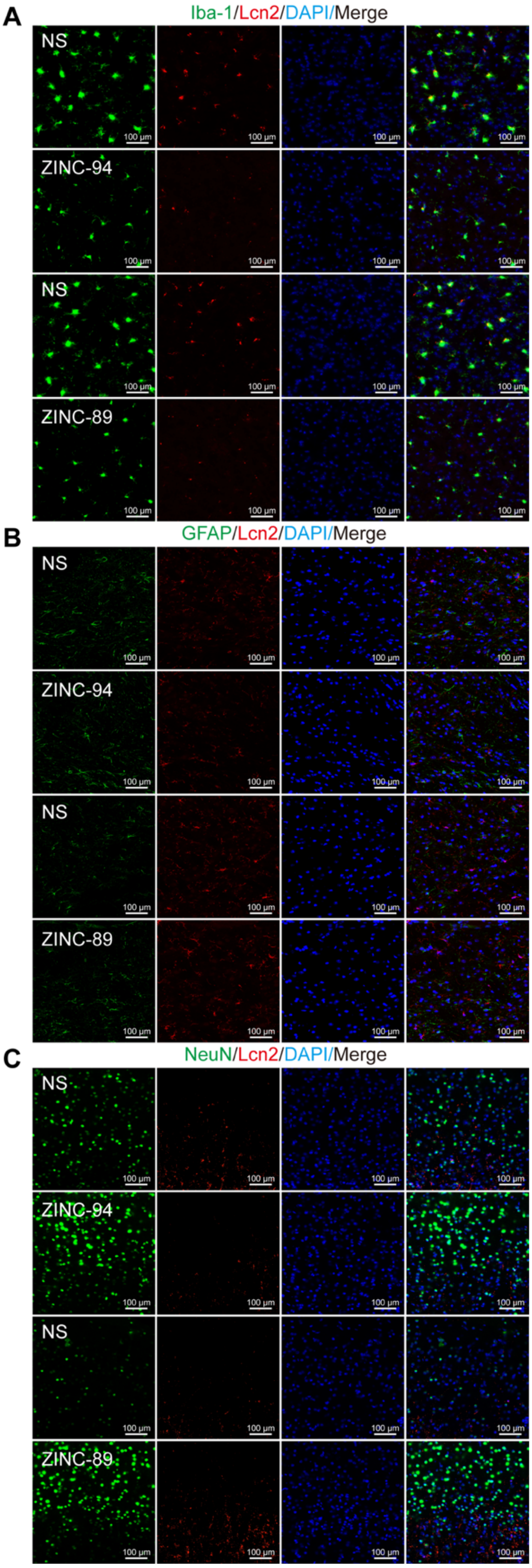

Figure S7. Co-localization levels of Lcn2 and various cell markers before and after inhibitor treatment.

A Co-localization levels of Lcn2 and the microglial marker Iba-1. **B** Co-localization levels of Lcn2

and the astrocyte marker GFAP. **C** Co-localization levels of Lcn2 and the neuronal marker NeuN.
